# The Metabolic Signature of In Vitro Produced Bovine Embryos Helps Predict Pregnancy and Birth after Embryo Transfer

**DOI:** 10.3390/metabo11080484

**Published:** 2021-07-27

**Authors:** Isabel Gimeno, Pablo García-Manrique, Susana Carrocera, Cristina López-Hidalgo, Luis Valledor, David Martín-González, Enrique Gómez

**Affiliations:** 1 Servicio Regional de Investigación y Desarrollo Agroalimentario (SERIDA), Centro de Biotecnología Animal, Camino de Rioseco 1225, 33394 Gijón, Spain; imgimeno@serida.org (I.G.); scarrocera@serida.org (S.C.); davydmg@hotmail.com (D.M.-G.); 2 Molecular Mass Spectrometry Unit, Scientific and Technical Services, University of Oviedo, Catedrático Rodrigo Uria s/n, 33006 Oviedo, Spain; garciampablo@uniovi.es; 3 Department of Organisms and Systems Biology, University Institute of Biotechnology of Asturias (IUBA), University of Oviedo, Catedrático Rodrigo Uria s/n, 33006 Oviedo, Spain; lopezhcristina@uniovi.es (C.L.-H.); valledorluis@uniovi.es (L.V.)

**Keywords:** bovine, embryo, metabolomics, mass-spectrometry, liquid-chromatography, dimethyl adipate, 12-hydroxydodecanoic acid

## Abstract

In vitro produced (IVP) embryos show large metabolic variability induced by breed, culture conditions, embryonic stage and sex and gamete donors. We hypothesized that the birth potential could be accurately predicted by UHPLC-MS/MS in culture medium (CM) with the discrimination of factors inducing metabolic variation. Day-6 embryos were developed in single CM (modified synthetic oviduct fluid) for 24 h and transferred to recipients as fresh (28 ETs) or frozen/thawed (58 ETs) Day-7 blastocysts. Variability was induced with seven bulls, slaughterhouse oocyte donors, culture conditions (serum + Bovine Serum Albumin [BSA] or BSA alone) prior to single culture embryonic stage records (Day-6: morula, early blastocyst, blastocyst; Day-7: expanding blastocyst; fully expanded blastocysts) and cryopreservation. Retained metabolite signals (6111) were analyzed as a function of pregnancy at Day-40, Day-62 and birth in a combinatorial block study with all fixed factors. We identified 34 accumulated metabolites through 511 blocks, 198 for birth, 166 for Day-62 and 147 for Day-40. The relative abundance of metabolites was higher within blocks from non-pregnant (460) than from pregnant (51) embryos. Taxonomy classified lipids (12 fatty acids and derivatives; 224 blocks), amino acids (12) and derivatives (3) (186 blocks), benzenoids (4; 58 blocks), tri-carboxylic acids (2; 41 blocks) and 5-Hydroxy-l-tryptophan (2 blocks). Some metabolites were effective as single biomarkers in 95 blocks (Receiver Operating Characteristic – Area Under the Curve [ROC-AUC]: 0.700–1.000). In contrast, more accurate predictions within the largest data sets were obtained with combinations of 2, 3 and 4 single metabolites in 206 blocks (ROC-AUC = 0.800–1.000). Pregnancy-prone embryos consumed more amino acids and citric acid, and depleted less lipids and cis-aconitic acid. Big metabolic differences between embryos support efficient pregnancy and birth prediction when analyzed in discriminant conditions.

## 1. Introduction

Accurate selection of competent in vitro produced (IVP) embryos for transfer to recipients is essential to maximize pregnancy and birth rates. Currently, the selection of viable IVP embryos is based on morphology and development stage, subjective criteria that rarely allow birth rates over 45% with either fresh or cryopreserved embryos [1,2,3,4,5]. The interest in identifying reliable markers of embryonic viability is higher in IVP, and mainly with respect to cryopreserved embryos, because of their intrinsic reduced viability to term. The lack of embryonic competence entails the use of more than usual numbers of embryos and recipients in order to achieve the planned born calf objectives, as well as more labor and farm inputs, thus leading to increased costs. Pregnancy biomarkers can lead to greater confidence in the preliminary embryo production and cryopreservation step prior to making the embryo transfer (ET) to recipients. Therefore, finding efficient markers of embryonic quality is a major objective for reducing both experimental and productive costs on farms.

In search of biomarkers, the individual quality of produced embryos has been assayed (i.e., predicted) by invasive and non-invasive approaches.

Biopsy is a typically invasive approach performed to collect embryonic cells or blastocoele fluid. Cell biopsy allows the study of chromosomal stability and expression of certain regulated genes linked to highly competent embryos [6,7,8,9]. With cell biopsy there is a risk of embryo damage that may lower pregnancy rates, leading to the misidentification of embryos with pregnancy potential, which may be analyzed as false negatives [7,9]. Biopsy for the extraction of blastocoele fluid seems to inflict less damage than cell removal [10], and metabolic profiles, proteins and genomic DNA are detectable in such fluid [11,12,13]. Biopsy requires great expertise, and, currently, no molecules have been identified in cattle blastocoele fluid that are associated with pregnancy. As minimal or no damage to each embryo is required in biomarker identification, non-invasive systems are potentially more advantageous than invasive systems. 

Non-invasive systems include time-lapse monitoring of embryo morphology and development kinetics, which has been reported to be effective in predicting pregnancy with sophisticated equipment [14,15]. Time-lapse can also be coupled with measurement of oxygen consumption, a non-invasive tool that can be used as a predictor itself [16,17]. A third type of non-invasive procedure aims to identify and quantify molecules released into and consumed from the CM by the embryo that can be associated with pregnancy success. Collection of CM from a single embryo culture step, immediately before any operation that the embryo would undergo within normal embryo culture (i.e., transfer or cryopreservation), does not impact embryonic viability. Thus, proteins, metabolites and the contents of extracellular vesicles (EV), such as small RNAs that the embryo releases, can be identified in CM [18,19,20]. Up to now, however, studies in CM of cattle embryos had described a metabolic fingerprint profile (Fourier Transform Infrared Spectroscopy) associated with pregnancy competence [21,22] or embryonic sex [23], but without identification of the molecules involved. The real association of molecules with the pregnancy potential of cattle embryo has only recently been shown in metabolites, by analysis of CM from vitrified/warmed (V/W) embryos by gas chromatography coupled with quadrupole time of flight mass spectrometry (GC-qTOF-MS) [24] and by electrospray ionization [25]. In our study, short-chain fatty acid (FA) metabolites were identified as candidate biomarkers predictive of birth [24].

However, other metabolomics techniques, such as Ultra High-Performance Liquid Chromatography (UHPLC), yielded a wider range and greater sensitivity than GC-qTOF-MS as untargeted platforms for metabolite identification and quantification [23,26,27,28]. To facilitate metabolomic studies of embryo CM, we developed a 24 h, single culture step (SCS) in synthetic oviduct fluid (SOF) w/o protein, which allows direct injection (i.e., without protein extraction) into the chromatograph, allowing for reliable metabolite identification for pregnancy prediction [24] and diagnosis of embryonic sex [26,27,28]. The SCS takes place as a last culture step, and it covers all embryonic stages comprised between compact morula and fully expanded blastocyst (FEB). Crucially, the SCS produces embryos with high viability after transfer as fresh, frozen/thawed (F/T) and V/W, and it can potentially be preceded by other embryo culture systems (e.g., serum and/or BSA containing) with or without group culture [1].

Interestingly, UHPLC-MS has not yet been used as a platform to investigate the pregnancy and birth potential of cattle embryos cultured in SCS. Nevertheless, the great complexity of factors that influence the bovine embryo metabolism in culture needs to be deciphered and overcome to reach positive results in metabolite biomarkers research. The morula to blastocyst is a very dynamic period in metabolic changes within early in vitro development [29,30]. In parallel to progress in blastulation, the embryo increases glycolytic flux and mitochondrial respiration [31,32] because of strict epigenetic control [33]. Such major changes are the tip of the iceberg in terms of the metabolic changes that arise when combined with breed, embryonic stage and culture conditions, whose certainly considerable impact has been evaluated in a companion study with UHPLC-MS during the development of morula and blastocysts in SCS [26]. Furthermore, the discrimination of embryonic stages has been shown to perfect pregnancy predictions in metabolomics [24].

We hypothesized that UHPLC-MS may be an efficient platform for discovery of metabolite pregnancy biomarkers with frozen and fresh IVP embryos. Together with a high variability in random factors, we included several fixed, controllable factors, supportive of a supervised strategy that improved predictions within embryonic stages, culture conditions, breed and cryopreservation status of the embryos.

## 2. Results

A total of *n* = 84 ETs were performed in 21 ET rounds. Supplementary Table S1 shows a summary of samples used in this study, while pregnancy and birth rates are shown in Table 1. 

**Figure 1:**
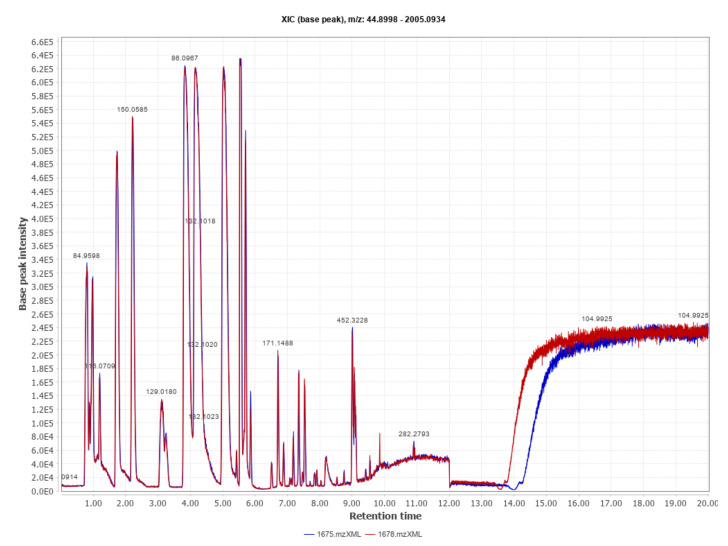
Metabolic spectral profile obtained by UHPLC-TOF MS-MS from two samples of embryo culture medium (CM) in positive-ion mode (range 100–1500 *m/z*). The total ion count (TIC) chromatograms correspond to a CM sample from one embryo diagnosed as pregnant (blue) and one embryo diagnosed as non-pregnant (red) on Day-40.

We obtained a total of 118,564 aligned spectral features which, after peak area processing, led to 6111 features retained. Sample signals were thereafter subtracted with their corresponding blank. The resulting output data were submitted to statistical analysis. Metabolic profiles typically obtained by UHPLC-TOF MS-MS are shown in Figure 1.

### 2.1. Multivariate Statistics for Sample Separation at Gestational Endpoints and Non-Random Factors

**Figure 2:**
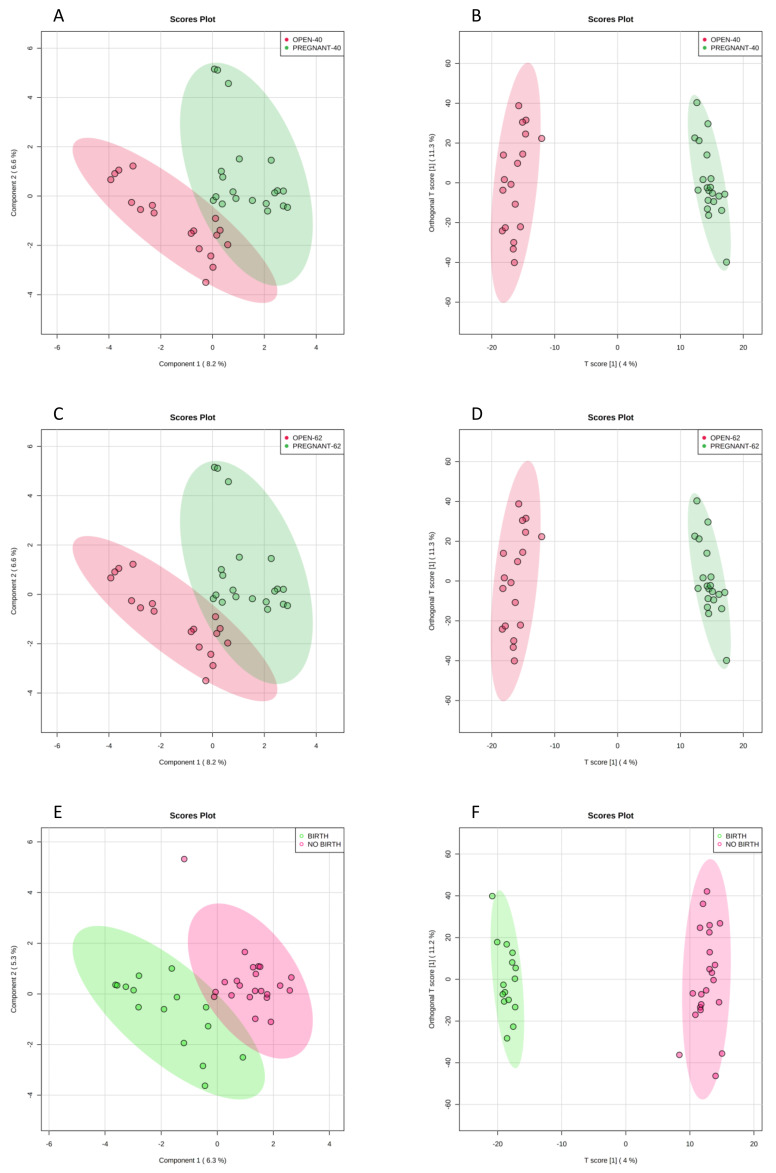
Separation of frozen embryos cultured in BSA by sparse partial least square-discriminant analysis (sPLS-DA) (**A**,**C**,**E**) and by orthogonal partial least square-discriminant analysis (OPLS-DA) (**B**,**D**,**F**) measured by gestational endpoints Day-40, Day-62 and birth, respectively. Figure 3B, Q2: *p* < 0.06, R2Y: *p* < 0.03. Figure 3D: Q2: *p* < 0.07, R2Y: *p* < 0.04. Figure 3F: Q2: *p* < 0.03, R2Y: *p* < 0.01. Empirical *p*-values were obtained after 100 permutations.

Multivariate analysis within the entire dataset with 6111 retained features did not yield significant sample separation by sPLS-DA and oPLS-DA at any pregnancy stage. Sample separation by including each of non-random factors (i.e., either breed, embryonic stage(s), culture or cryopreservation) did not lead to significant sample separation either. Using combinations of two factors, however, we identified differences when sample separation was performed by culture condition and cryopreservation. Thus, frozen embryos cultured in BSA showed clear separation by sPLS-DA (Figure 2A,C,E) and significant separation by OPLS-DA (Figure 2B,D,F) at gestational endpoints Day-40, Day-62 and birth, respectively.

**Figure 3:**
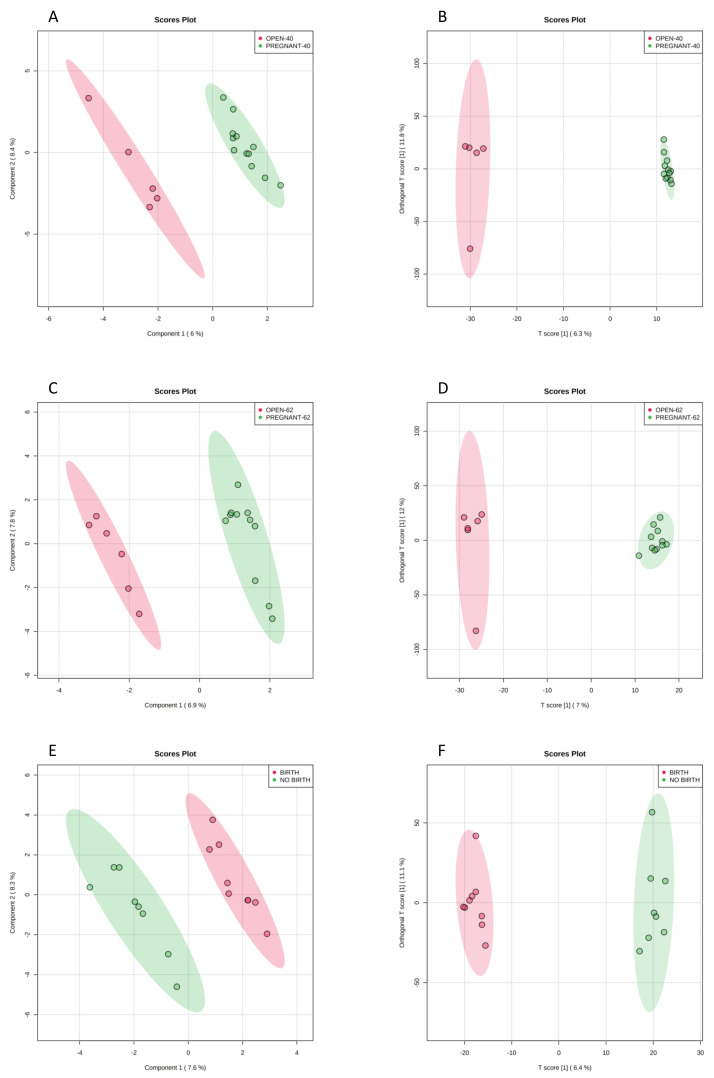
Separation of fresh embryos cultured with BSA was predictive of pregnancy at Day-62 (**C**,**D**) (Q2: *p* < 0.07, R2Y: *p* < 0.04), although not for pregnancy at Day-40 (**A**,**B**) and birth (**E**,**F**) (Q2 and R2Y > 0.10). Empirical *p*-values were obtained after 100 permutations.

Interestingly, this separation included both Day-7 and Day-8 embryos and represented the largest di-factorial dataset (*n* = 38 samples) in the multivariate study. On the contrary, embryos with FCS (*n* = 18 samples frozen, and *n* = 11 samples fresh), did not show clear discrimination at any endpoint. However, fresh embryos cultured with BSA (*n* = 17) were predictive of pregnancy at Day-62 by sPLS-DA and OPLS-DA (Figure 3C,D), although not at Day-40 (Figure 3A,B) and birth (Figure 3E,F). No other combination with two or more factors was significant for discrimination by multivariate analysis.

### 2.2. The Bull: Influence of a Random Factor

**Figure 4:**
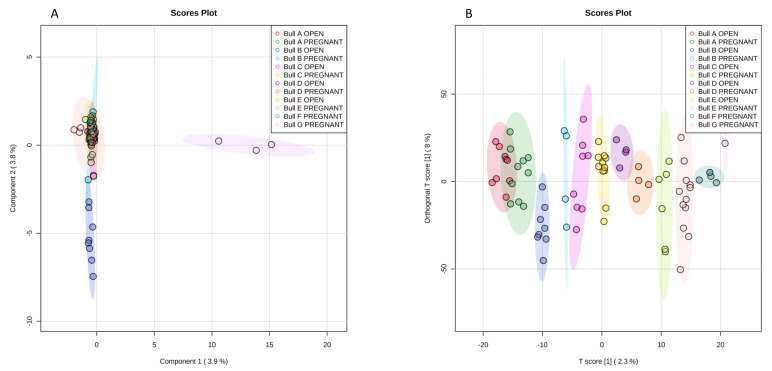
Separation of pregnant and non-pregnant samples from 7 different bulls as a function of pregnancy on Day-62, by sPLS-DA (**A**) and by OPLS-DA (**B**) (Q2: *p* < 0.01, R2Y: *p* < 0.01; 2 samples of Bull G-pregnant hidden down the legend). Empirical *p*-values were obtained after 100 permutations.

We analyzed the individual bull (*n* = 7) as a random factor by multivariate analysis as a function of pregnancies on Day-62. Bull data were not analyzed at birth because one bull had only two birth samples, one bull had only two no-birth samples, and a third bull lost one recipient by sudden death after Day-62. Between-bull sample separation was not evident by sPLS-DA, with just Bull B-open and Bull G-pregnant outside of a narrow cluster formed by the remainder groups (Figure 4A). Separation by single bull and pregnant status was nevertheless more evident and significant within OPLS-DA (Figure 4B); interestingly, overlap between bulls and type of samples was not observable, suggesting that precluding individual variability (i.e., use of a single bull in biomarker studies) can hide the identification of metabolites that would act as biomarkers.

### 2.3. Block Analysis with Fixed Factors to Identify Metabolite Biomarkers

The multi-factorial combination of the 6111 retained metabolite signals led to obtaining 17,331 blocks starting from metabolite signals defined by combining the fixed factors controllable in the laboratory, i.e., bull breed, culture medium, cryopreservation, embryonic stage at the onset of single culture (Day-6 or Day-7) and embryonic stage at the end of single culture (Day-7 or Day-8). Of those, 3946 blocks contained metabolite signals predictive of pregnancy at Day-40, Day-62 and birth. Metabolite identity was explored at this stage, and only those metabolites with a compound mass below 10 ppms to their exact mass, and also with at least three MS2 ions, were considered to have been confidently identified (see Materials and Methods and Supplementary Table S2).

After metabolite identification, the study yielded 511 blocks with 34 significantly accumulated metabolites (frozen and fresh), 198 for birth, 166 for Day-62 and 147 for Day-40 (Supplementary Table S3). Log FChs in metabolite relative concentrations expressed in all tables and figures are shown as pregnant/non-pregnant ratio. In their majority, Log FChs can be considered as qualitative (e.g., when >│99.000│), with higher abundance of metabolites in CM from non-pregnant recipients (positive FCh: 51; negative FCh: 460). The value of a metabolite as a biomarker was therefore given both by its ROC-AUC value > 0.700 and by the proportion of pregnancies correctly predicted under the embryo culture conditions of each block. Of 34 significantly accumulated hits, 33 metabolites were represented in blocks of frozen embryos (with 16 metabolites of frozen embryos not represented within fresh embryos); 17 metabolites were in blocks independent of embryo cryopreservation; and only one block was exclusive of fresh embryos (5-Hydroxy-L-tryptophan) (Supplementary Table S3). Three metabolites had more blocks at birth within fresh than within frozen embryos (L-glutamic acid, L-lysine and phenylacetaldehyde). As reviewed in our published database for sex analysis within developmental transitions [26], eight metabolites present in 15 blocks were significantly affected by embryonic sex. 12-Hydroxydodecanoic acid was the metabolite most affected by sex, with 5/43 blocks involved. As Supplementary Table S3 is large and contains blocks that partially overlap, the most relevant information was extracted to be shown in smaller tables through the main text. 

**Figure 5:**
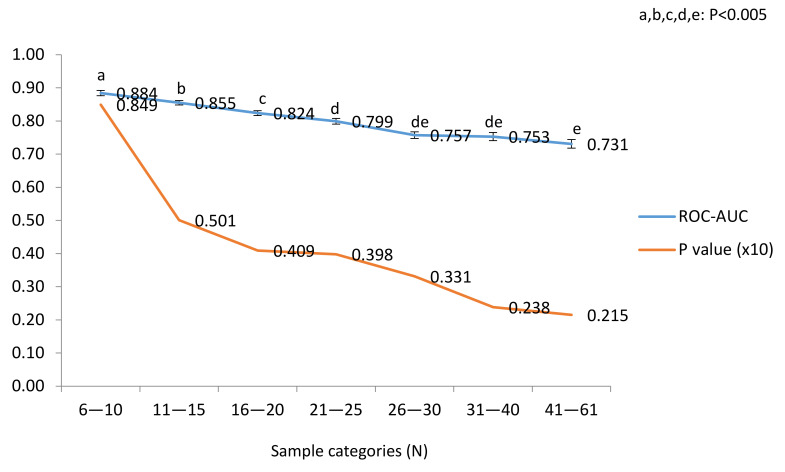
ROC-AUC and their *p*-value averages plotted by categories of sample numbers (Pearson’s correlation coefficients are shown). *p*-values given in a ×10 basis for scale consistency with ROC-AUC. Data from *n* = 511 blocks distributed into 78, 131, 106, 85, 52, 30 and 29 blocks from lower to higher sample categories.

The overall performance within the selected blocks under study is shown in Figure 5. 

Average ROC-AUC values (LSM ± SEM) decreased with sample numbers, but their significance (*p*-values, shown in a ×10 basis for consistency of scale) increased. Negative correlations were recorded between ROC-AUC and sample numbers (R: −0.6086; *p* < 0.0001) and between *p*-values and sample numbers (R: −0.34114; *p* < 0.0001). Interestingly, the ROC-AUC slope was attenuated in categories above >21 samples, close to the plateau ROC-AUC ≈ 0.700 and within the number of samples covered in this study.

### 2.4. Univariate Statistics with Candidate Biomarker Metabolites

**Figure 6:**
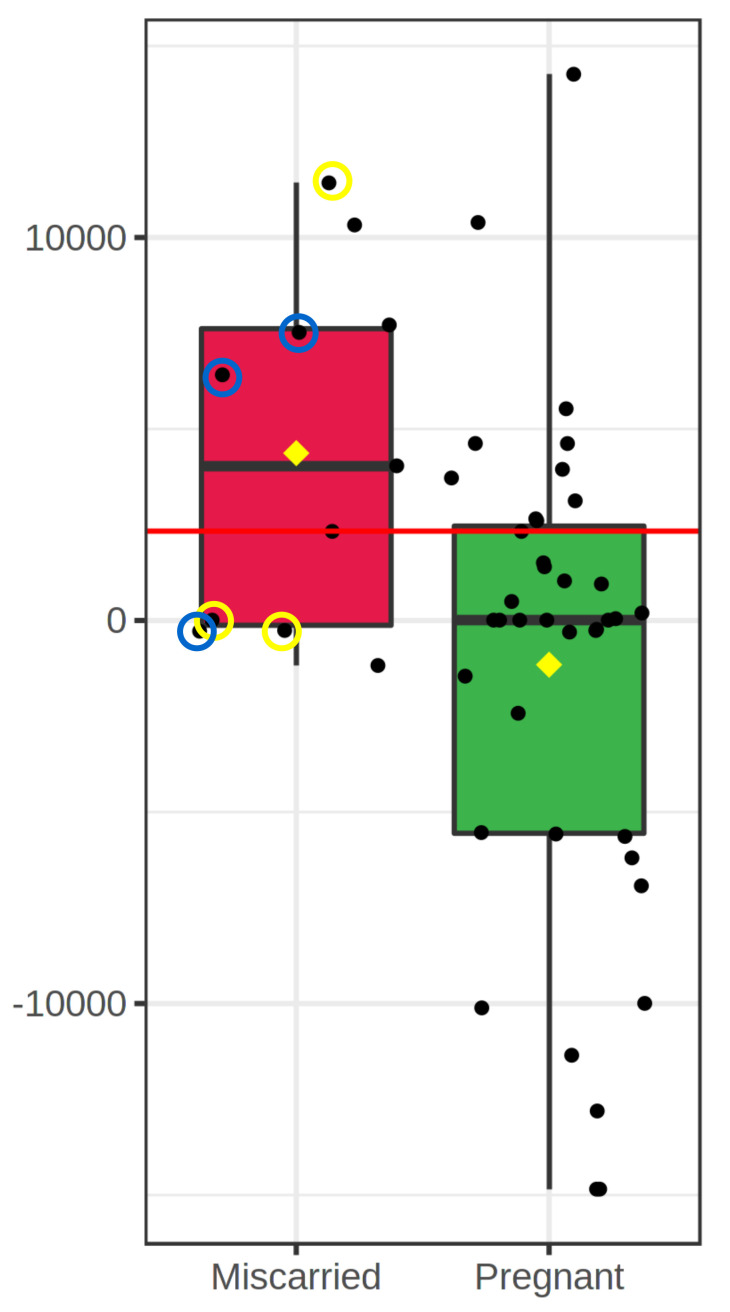
Boxplot for Methyl adipate concentrations that differ between embryos that miscarried after Day-40 and embryos that led to pregnancy on Day-40. Circled samples indicate fresh embryos cultured in BSA (yellow line), and frozen embryos cultured with FCS (blue line); non-circled samples are frozen embryos cultured with BSA (no miscarried samples from fresh embryos cultured with FCS).

The metabolites previously identified in blocks were analyzed for pregnancy endpoints in the entire dataset using a GLM model that included all effects identified (cryopreservation, culture, bull breed, embryonic stages—0 h and 24 h—and embryonic age), and a Bonferroni correction (*p* < 0.10) as a false discovery rate test (Table 2).

l-Lysine was the only metabolite that significantly differed between pregnant and open recipients at the three pregnancy endpoints (Day-40, Day-62 and Birth), while l-Leucine, Palmitoylethanolamide and Lauroyl diethanolamide differed for predicting birth but not for earlier endpoints. On the contrary, concentrations of l-Valine, Dimethyl adipate and Phosphatidylethanolamine (18:2/20:2) changed as a function of early pregnancy endpoints, but not birth. Miscarriages that occurred after Day-40 were *n* = 11 cases; interestingly, embryos that experienced such late miscarriage differed in their levels of Dimethyl adipate (discriminated both pregnancy to term embryos vs. late miscarriage—Figure 6—and vs. open embryos; *p* = 0.0011).

Class metabolite analysis in all identified blocks. Metabolites were grouped into five taxonomical classes identified in total blocks at the three developmental endpoints (Table 3): Class (1) Lipid and lipid-like molecules (224 blocks—the most predictive class; 12 metabolites); Class (2) amino acids (186 blocks; 15 metabolites); Class (3) benzenoids (58 blocks; 4 metabolites); Class (4) Tricarboxylic acids (41 blocks; 2 metabolites); and Class (5) Tryptamines and derivatives (2 blocks; 1 metabolite) (metabolite subclasses are defined in Supplementary Table S2).

All lipids identified contained or were fatty acids (FA), and/or had a role in the FA metabolism. Interestingly, all amino acids (except pipecolic acid) and citrate were components of the SOF formula used. The abundance of predictive blocks increased with gestational endpoint, being maximal for birth (198 blocks), intermediate for Day-62 (166 blocks), and lowest for Day-40 (147 blocks). This increasing abundance was mainly observed through Classes 1, 2 and 3. As counted by embryo cryopreservation, frozen embryos recorded 309 blocks, and fresh embryos 51 blocks, while 151 blocks were independent of cryopreservation. The following metabolites had outstanding representation in blocks: 12-Hydroxydodecanoic acid (43 blocks) and methyl adipate (41 blocks), followed by cis-Aconitic acid, Linoleamide, phosphatidylethanolamine(18:2/20:2), Lauroyl diethanolamide, p-Cresol, MG(16:0/0:0/0:0), and pyroglutamic acid, with 29 to 20 blocks each.

### 2.5. Pregnancy Endpoint Analysis

#### 2.5.1. Overview

Relevant predictions from early pregnancy stages (i.e., Day-40 and Day-62) were drawn from factors analyzed without using embryo cryopreservation and breed as factors. However, contrary to earlier diagnosis endpoints, accurate birth predictions required discrimination by cryopreservation stage (i.e., frozen vs. fresh), which was improved when combined with breed, culture conditions or both. In contrast, single embryonic stages showed lower ROC-AUCs than cryopreservation, breed or culture, although stages greatly improved birth predictions for all these latter factors (as observed in Supplementary Table S3). The discordance between early and late pregnancy predictive block patterns reflects a different late pregnancy course between fresh and frozen embryos, with marked metabolite differences, as seen also between embryos that led to miscarriage, failure of embryos to set pregnancies on Day-40 and pregnancies to term.

#### 2.5.2. Impact of Biomarkers through Pregnancy Endpoints

**Figure 7:**
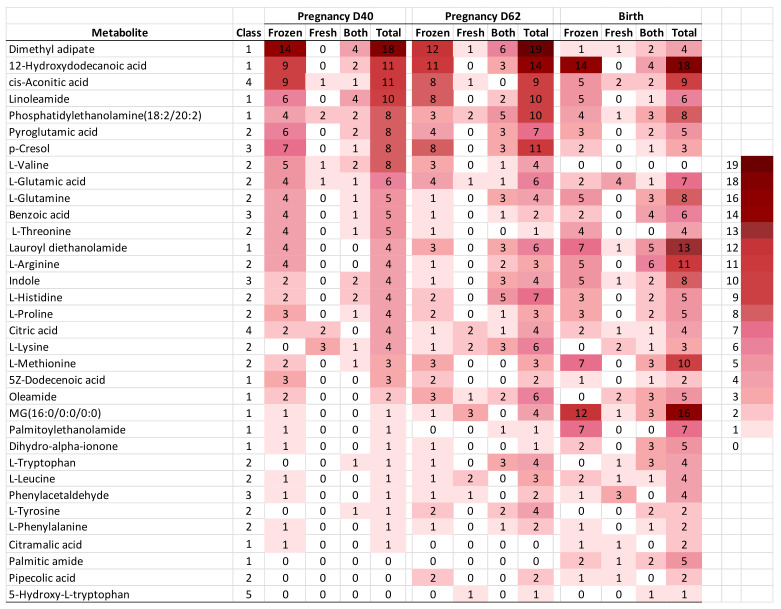
Heatmap representative of abundance of blocks within metabolites that predicted pregnancy at the developmental endpoints D-40, D-62 (predictive blocks shown correspond to fresh and frozen, as well as non-cryopreservation dependent, embryos). The number of blocks per metabolite is ranked by the column “Total” within blocks at Pregnancy Day-40. Taxonomical classes (Class): (1) Lipids; (2) Amino acids; (3) Benzenoids; (4) Carboxylic acids; (5) Tryptamines and derivatives.

The tracking of the impact of each biomarker through the developmental endpoints is depicted as a heatmap in Figure 7.

Metabolites were ranked by the number of “Total” blocks that were predictive at Day-40. The color scale accounts for the numbers of blocks for each metabolite through pregnancy endpoints with frozen and fresh embryos, blocks independent of cryopreservation, and total number of blocks. Generally, the arrangement by total blocks at Day-40 showed abundant metabolite blocks at later pregnancy endpoints (mainly in the top of Figure 7), in particular among the most abundant frozen over fresh embryos. Low abundance metabolites in Figure 7 at Day-40 are at the bottom, in a region that shows increasing abundance of metabolites through pregnancy endpoints, with contrasting metabolites that predicted at birth but not earlier (i.e., MG(16:0/0:0/0:0), indole and palmitoylethanolamide, as relevant metabolites). On the contrary, other metabolites displayed no or lower predictive ability at birth, but higher block numbers for Day-40 and/or Day-62 (i.e dimethyl adipate, pyroglutamic acid, linoleamide, p-cresol and l -valine). Interestingly, dimethyl adipate was strongly involved in miscarriage (see above “Univariate statistics within candidate biomarker metabolites”), and accounted for the most striking differences between abundant blocks on Day-40 (18 blocks) and on Day-62 (19 blocks), as compared with blocks at birth (4 blocks); such differences were more pronounced with frozen embryos. In contrast, Lauroyl diethanolamide, the other metabolite involved in miscarriage, showed increasing numbers of total blocks through Day-40, Day-62 and birth (i.e., 4, 6 and 13 blocks).

### 2.6. Single Biomarker Metabolites Predict Pregnancy with >70% Effectiveness

Hits bearing ROC-AUC > 0.700, with which it is possible to accurately predict pregnancy in specific blocks with ≥70% effectiveness as single metabolites, are shown in Table 4.

This selection consisted of 95 blocks, all independent from the embryonic stage at the onset of individual culture (0 h), and only 6 blocks in which fresh embryos were dependent on embryonic stage at 24 h (end of individual culture). This dependence at 24 h meant the exclusion of two embryos (one early blastocyst and one blastocyst) transferred in the fresh dataset, and one block with FEB fresh embryos. Therefore, insofar as ExB and FEB are involved, the embryonic stage generally did not influence the predictions with single metabolites shown in Table 3. Birth was once again the most predictive endpoint (37 blocks), followed by 29 blocks for Day-62 and 28 blocks for Day-40. Twenty blocks predicted with fresh embryos, while 74 blocks predicted with frozen embryos, and 1 block was independent of cryopreservation (indole). By breed, 73 blocks predicted within AV and eight in Holstein; while 13 blocks were independent of breed. Class summary reflects 23 blocks with nine metabolites (lipids); 46 blocks with 11 metabolites (amino acids and derivatives); 15 blocks with four metabolites (benzenoids); and 12 blocks with two metabolites for carboxylic acids. The following top 11 hits predicted with 90% to 100% accuracy: Lauroyl diethanolamide, l-Glutamic acid, l-Proline, l-Methionine, Pyroglutamic acid, l-Glutamic acid, l-Arginine, l-Lysine, l-Threonine, l-Glutamine, l-Methionine, all belonging to the amino acid and derivatives class, except for one lipid.

### 2.7. Combinations of Biomarker Metabolites Increase Pregnancy Prediction Rates

We combined the predictive power of single metabolites to obtain overall predictions >0.800 within blocks and series. A series is defined by the same conditions for cryopreservation, breed and culture, supported by one or more developmental IC-stages. Metabolite combinations generally (but not in all cases) gave higher predictions for larger data sets than did single metabolites. A general list of predictive-compliant series (17) with their blocks and metabolites that can be used for combinations is shown in Supplementary Table S4. For clarity, the best combination from each predictive series is shown in Table 5.

Overall combinations (Supplementary Table S4) were more informative for birth (9 series; 89 blocks), and decreased towards Day-62 (5 series; 56 blocks) and Day-40 (3 series; 45 blocks). By classes, lipids were the most represented in blocks (90), followed by amino acids (68), benzenoids (17), carboxylic acids, (13) and tryptamines and derivatives (1). Dimethyl adipate (18), 12-Hydroxydodecanoic acid (17), linoleamide (13), pyroglutamic acid (9) and cis-aconitic acid (9) were the most represented metabolites. This representation is consistent with the general relevance of each single metabolite. Interestingly, nine series (88 blocks) were independent on cryopreservation, the remainder being 83 blocks for frozen embryos (five series) and 18 blocks for fresh embryos (three series). Culture showed nine independent series (103 blocks), with BSA and FCS being necessary in six series (75 blocks) and two series (11 blocks), respectively. Combined predictions showed great independence of bull breed (14 series), and only the Holstein breed was required as a factor (three series; nine blocks), with AV breed not being necessary. Collectively, efficient predictions can be made with little or no dependence on fixed factors. Thus, as shown in Table 5, predictive information was obtained from very basic conditions (i.e., independent of cryopreservation, culture and bull) at the three endpoints with three (Day-40), four (Day-62) and five (birth) combined metabolites (as seen in series 3, 8 and 17, respectively). On the contrary, 11 series with only two combined metabolites each required only one or more conditions to be predictive, as shown with series 4 and 5, showing combinations of three and four metabolites on Day-62, and series 15 with all metabolites at birth. Interestingly, whatever the path, all original metabolites shown in Supplementary Table S4 can form combinations resulting in predictive values >0.800 and, in some cases, also reaching the highest predictive values summarized in Table 5.

### 2.8. Validation

Biomarker validation: The strong random and pre-planned variability with which our study was designed through different block studies ensures a principle of proof validation (different populations, samples and conditions). Thus, we based the relevance of biomarkers primarily on the numbers of blocks in which they participate with ROC-AUC > 0.700, either singly or combined, and, for practical purposes, on the total number of embryos whose pregnancy probability was correctly identified (as a function of ROC-AUC and frequency of appearance of the embryos in question within each block).

## 3. Discussion

In this study, we used fresh and frozen IVP embryos transferred to recipients to non-invasively identify high numbers of metabolite biomarkers that predicted pregnancy in spent CM.

Thus, multivariate analysis pointed out that supervision with at least two factors favors discrimination between pregnant and open recipients, focused on the fresh and frozen BSA datasets. Interestingly, the frozen dataset included Day-7 and Day-8 embryos, suggesting that quality profiles of competent embryos do not differ with age or retarded development. Rather, the lower survival of Day-8 embryos [5,24,34] could be due to lower rates of high-quality embryos that essentially do not differ in metabolism from Day-7 counterparts. The bull was the more powerful effect identified (in line with 26), with almost complete separation of single bulls and pregnancy status (given as a function between metabolite signals and pregnancy on Day-62). This random bull effect must be counteracted with individual variability (seven bulls in our study), since the way to discriminate biomarkers (e.g., higher ROC-AUC, high FCh, and statistical tests with the lowest *p*-values) is hierarchical, and therefore highly dependent on specific bull interactions (i.e., a potential risk of misidentification with a single bull). In our study, we identified abundant numbers of metabolites that predicted pregnancy at the three stages diagnosed. Herein, smaller sample datasets with fixed factors (i.e., blocks) helped to discover metabolite biomarkers, as hypothesized. In contrast, in our previous study with lower sample numbers to accomplish a factorial study (*n* = 36 V/W embryos from Holstein and AV) [24], embryos were clustered together by discriminant analysis. The present analysis (*n* = 84 embryos), nevertheless, did benefit from discriminant factorial strategies, as many more biomarkers were obtained.

The number of predictive metabolites and blocks was higher in frozen than in fresh embryos, which is not surprising, because the number of samples in the frozen dataset was approximately double that in the fresh dataset. The interest, within the field of biomarker studies, is potentially higher in frozen embryos, as their birth rates are lower than fresh embryos (i.e., more added value is expected), and international exchanges of high-cost, genetic-merit embryos are made with cryopreservation. However, more than a few metabolites qualified both for fresh and F/T embryos, suggesting that common predictions are possible. Thus, metabolites with different representation between frozen and fresh embryos could shed light on the metabolic facts than make them different. Among these, the biggest differences were recorded for birth within 12-Hydroxydodecanoic acid, lauroyl diethanolamide, l -Methionine, MG (16:0/0:0/0:0) and palmitoylethanolamide, and, at Day-40 and Day-62, dimethyl adipate, 12-Hydroxydodecanoic acid, cis-aconitic acid, linoleamide and p-Cresol. Again, this greater representation of lipids is in agreement with our recent study with V/W embryos [24]. The only metabolite not represented exclusively in frozen embryos (5-Hydroxy- l -tryptophan) had low incidence, being present in two blocks. Interestingly, the low impact of embryonic sex on specific developmental transitions, metabolites and blocks, confirmed in a sex-specific larger dataset [26], permitted us to discard sex as a possible confounder for pregnancy prediction (consistent with [25]). The scarcity of metabolites with sex dependence within blocks facilitates pregnancy prognosis. Independence of sex is interesting, even with sex-sorted spermatozoa, since the efficiency of sex sorting is high but not 100% [35]. The vast amount of information obtained in our study was unexpected, given the limited information in previous studies analyzing metabolomic biomarkers in CM for pregnancy or even for short-term in vitro developmental endpoints [24,25,36].

Testing single metabolites with ROC-AUC > 0.700 and/or multiple combinations of metabolites with ROC-AUC > 0.800 took advantage of a variety of factors to achieve higher pregnancy predictive coverage of embryos that are metabolically diverse. Difficulties in obtaining a single biomarker predictive of embryo development have been cited [36], and are consistent with recent studies [24,25]. Fortunately, the factors selected (breed, cryopreservation, stages, and culture supplements) are under normal control in an IVP laboratory, and new agreement and validation studies are needed to refine which of the identified candidate biomarkers are finally effective. Dairy, beef and crossbred cattle breeds markedly differ in metabolism [37,38,39]. Although breed-linked metabolism does not have an extensive influence on early embryos [24], metabolomic differences measured in embryo CM yield breed-specific (dairy and beef) changes in certain metabolites [26]. Culture systems are also potential sources of metabolic variation, as oxygen tension and culture composition (glucose, culture with FCS and BSA) can induce changes in the carbohydrate, lipid and amino acid metabolism in embryos [31,40]. However, although testing the large assortment of laboratory culture conditions is impractical, the use of a common analytical SCS (24 h), which is highly tolerant with embryonic competence, can led to efficient inter-laboratorial biomarker identification. Thus, failure to identify the abundance and/or consistency of cattle biomarkers in previous studies could be attributable to lower sample data sets, no incorporation of specific factors or lack of normalizing conditions.

The breed bulls in our study are used worldwide (Holstein), while the AV is a double-muscled cattle breed comparable to Belgian Blue, Piamontese and other commercial beef breeds; therefore, our general findings and those in AV have wide impact. Metabolic differences imposed by individual bull (random) and breed bull (fixed) effects (with 20 and 15 metabolites affected, respectively; [26]) suggest counteracting random variability in biomarker studies using sufficient bulls (seven in our study). Our study also lacks some stage transitions with low incidence in culture and/or shows unbalanced pregnant and open samples (e.g., in culture with FCS, Day-6 morulae develop to Day-7 at very low rates and with low viability). We should also confront our results within recipients evaluated for their pregnancy competence [24]; the information obtained from embryos could be obtained with an even greater precision and confidence, and with wider prediction ability. The adequacy of our procedures, with blocks showing increased statistical significance value above the 21-sample threshold, suggests appropriate predictive consistency with stability over the value ROC-AUC = 0.700.

Amounts of predictive blocks were higher for birth and decreased through earlier pregnancy stages, as shown with previous studies with fresh [21] and V/W embryos [21,24]. In contrast, superior-quality in vivo embryos collected from FSH-superstimulated cows that were cultured in vitro for 24 h showed higher predictive ability by FTIR on Day-60 than at birth [22]. Such differences could entail a differential signature for embryo losses (between Day-60 and birth) present within IVP embryos, not within in vivo embryos. In this regard, biomarkers that differed in their abundance between pregnancy endpoints could be predictive of specific embryo developmental competence. Miscarriage is a difficult topic to investigate because of obscure etiology, low incidence, and the influence of conditions such as cryopreservation and the presence of serum [3]. The cases of dimethyl adipate (and, to a lesser extent, p-cresol), highly pregnancy predictive for Day-40 and Day-62, but with low predictive ability at birth, are consistent with their pregnancy and miscarriage identification in the general dataset. The effects of dimethyl adipate on reproductive organs are unknown. However, in rats, the compound may induce heat shock protein [41], although it is not known as teratogenic [42]. A contrasting pattern was shown by other metabolites, with MG (16:0/0:0/0:0) and palmitoylethanolamide being relevant, and predictive for birth in many blocks in frozen embryos, but not for earlier pregnancy endpoints. Such metabolites were not involved in general miscarriage, but they could inform of such differences in frozen embryos. Interestingly, the abundant presence of lipids as biomarkers is consistent with our former study wherein V/W embryos that did not reach pregnancy to term released higher amounts of non-esterified saturated FA (NEFAs) (stearic, capric and palmitic acids) and glyceryl-monostearate into the CM [24]. In the present study, all lipids identified were FAs or FA derivatives, with palmitic acid being present through many compounds (i.e., Phosphatidylethanolamine (18:2/20:2), palmitic amide, palmitoylethanolamide), an indication that the lipid stock and its breakdown determine embryonic quality. Interestingly, palmitic acid concentrations are higher within the less viable IVP than in vivo developed embryo [43], and FCS increases lipid contents and palmitic, palmitoleic, oleic and stearic acids [44] and reduces tolerance of embryos to cryopreservation [45,46,47]. In domestic species, lipids are stored in blastomeres as trygliceride [48,49] with higher stocks in IVP embryos than in vivo collected embryos [43]. Lipid granules decrease through blastulation concurrent with an increase in lipolytic gene expression [40,50,51]. The lipid breakdown in embryos is consistent with the embryonic ability to develop in a medium deprived of exogenous substrate [52], and lipid granules decrease in our protein-free SCS [40].

IVP embryos show more oxidized but scarce glycerophospholipids, a group of membrane constituents. Slow freezing alters glycerophospholipids in in vivo and IVP embryos [43] (lysophosphatidylcholines). Phosphatidylcholines are methylated phosphatidylethanolamines whose increase in membranes enhances embryo survival to cryopreservation [53]. In our work, phosphatidylethanolamine(18:2/20:2) had, however, a comparable predictive impact through frozen and fresh blocks. Palmitoylethanolamide accumulates during cellular stress, and it can counteract cellular stress and inflammation [54], and is found in amniotic and other reproductive fluids [55].

An excess of palmitic and other FAs impacts DNA methylation, therefore leading to epigenetic alteration, as observed within different cell types [56], and in fasting to post-prandial transitions in obese humans [57,58]. These events are conceptually similar to the dynamic reprogramming during embryonic development, by which environmental stimuli (including assisted reproductive technologies) lead to acquisition of a stable, modified genotype later in life [59] and, in this case, loss of pregnancy competence. NEFAs are mainly responsible for low embryonic fitness [24,60,61,62,63]. Our results confirm findings with V/W embryos [24] in which fully viable embryos were those with less active lipid catabolism, as shown by reduced FA contents in their CM.

Although we sought a very active lipid metabolism generally associated with non-pregnancy-prone embryos, embryos that led to pregnancy and birth incorporated more amino acids. Therefore, a more active amino acid metabolism could be supportive of pregnancy, which is in agreement with a recent study [25]. Endogenous FA and amino acids can be used as nutrients by early embryos, but it is unclear whether our results match with an efficient use of metabolic resources by the most viable embryos, as postulated [64]. The use of amino acids as markers of embryo development was proposed in human embryos by Houghton [65]. Actually, embryos from different species, development stages, quality, morphology and sex differ in their profile of amino acid consumption [27,28,65,66,67,68,69,70,71,72,73,74]. Such metabolic complexity can distort the identification of viable embryos, hence the limited pregnancy predictive metabolomic studies in bovine embryos and the lack of molecular specification [21,22,24,25]. Most amino acid blocks showed negative FCh (163 blocks) vs. 23 blocks with positive FCh, reflecting higher amino acid intake in embryos that reached pregnancy and birth. Additionally, as with lipids, most blocks showed large FCh (qualitative) differences, typical within the exogenous protein-free CM we used over BSA containing CM [28].

Amino acids had a relevant presence as single biomarkers (23 out of 24 top ROC-AUC > 0.90 were amino acids and derivatives), as essential (l -Methionine, l -Arginine, l -Lysine, l -Threonine) and non-essential (l -Glutamic acid, l -Proline and l -Glutamine), together with the amino acid derivative Pyroglutamic acid. Within combinations of biomarkers, amino acids (20 hits) had a presence comparable to lipids (19 hits). A recent ESI-MS study identified higher depletion of glutamic acid by embryos that led to pregnancy on Day-60 [25]. Glutamic acid was present in 19 blocks, seven of them birth-predictive. Such authors also identified high pyruvate and low lactate as being predictive of pregnancy at Day-60 in a cohort of FEB embryos. We identified a regulated metabolite with mass corresponding to lactate, whose identification did not reach our standards (not shown); it had negative FCh in four blocks, consistent with [25].

Benzenoids were present in 58 blocks; 53 with negative and five positive FCh. Benzoic acids derivatives (e.g., hippuric acid) were the second most abundant subclass of metabolites present in the cow uterine fluid (UF) in the cycle days 0 and 5 [75]. Thus, P-cresol, an end-product of protein degradation which could act as a signaling metabolite, peaks in UF on Day-5 and decreases onwards [75]. Phenylacetaldehyde is an oxidation-related aldehyde that may form from styrene [76]; styrene is used in laboratory plasticware. Indole is a potent antioxidant. Tryptophan is an indole derivative and the precursor of the neurotransmitters serotonin and melatonin (N-acetyl-5 methoxytriptamine). However, indole itself is a microbial metabolite, while 5-Hydroxy- l -tryptophan had a low representation in our study (two blocks), suggesting an unclear role. The Krebs cycle metabolites citric acid and cis-aconitic acid also decreased in CM from pregnant recipients. Citrate is a constituent of our SOF medium.

Our findings with amino acids, tri-carboxylic acids and FA metabolism, together with the influence of certain lipids on membrane function, can be integrated with mitochondrial function and morphology, as representative of the success of the embryonic viability [77]. Embryos with true pregnancy potential show active amino acid and citric acid intake, but lower lipid metabolism and lower depletion of cis-aconitic acid. Lower lipid breakdown corresponds to less release of lipids into CM; such embryos incorporate citrate for oxidative metabolism, which follows the Krebs cycle through cis-aconitic acid, which would be used and therefore non-excreted, as opposed to non-pregnancy-prone embryos. We suggest that less competent embryos accumulated more lipids in their cells.

The block design and the metabolic complexity observed between embryonic stages, under the influence of breed, culture conditions and ultimately cryopreservation, made it not possible to provide either a pathway study or a general in-depth description of the metabolic changes between embryonic transitions. Embryologists may check whether specific biomarkers identified in our work fit well with their particular embryo culture, breed and cryopreservation conditions. We consider it a practical advantage that several amino acids, citric acid and cis-aconitic acid from the embryo CM have analytical kits available in the market. The same occurs with some lipids identified (e.g., linoleamide and dodecenoic acid). Progress in refining pregnancy predictions is expected when more studies and trials become available.

An unexpected number of metabolites were predictive of pregnancy and birth under contrasting conditions. There was, however, a window for single biomarkers, and a larger window for combinations of biomarkers. Cryopreserved and fresh embryos share pregnancy and birth biomarkers, although many were specific to fresh or frozen embryos. Discovering embryonic biomarkers for pregnancy and birth is possible under the control of developmental stage, culture conditions and breed, as traits that make embryos differ in their metabolism. The information on the pregnancy predictive capacity of the metabolome in CM from cattle IVP embryos is novel, and selecting appropriate metabolites for targeted research work in particular laboratorial conditions is possible.

## 4. Materials and Methods

**Figure 8:**
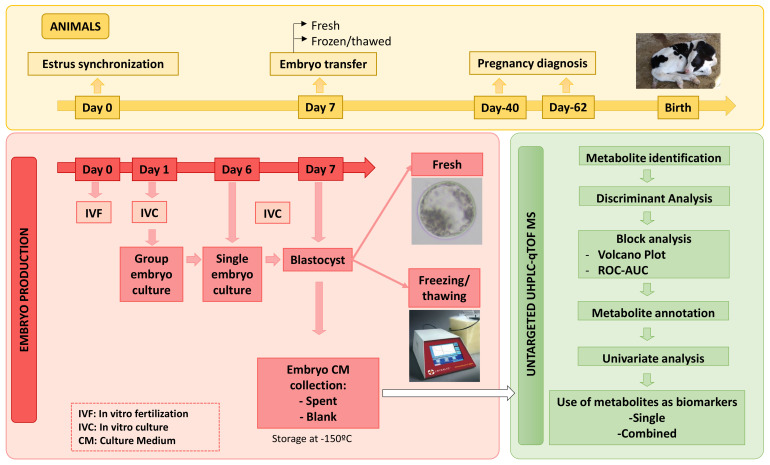
General experimental workflow.

All reagents were purchased from SIGMA (Madrid, Spain) unless otherwise stated. Figure 8 describes the experimental procedures workflow.

### 4.1. Rationale

In this work, we described the conditions whereby metabolites can perform as biomarkers within in vitro embryo culture. The predictive value of metabolites is not only defined by specific ROC-AUCs, but also, from a practical point of view, the incidence of particular embryo stages for which a metabolite is predictive must be defined within a specific culture system.

This study analyzed the metabolomic profiles in CM from F/T and fresh (control) embryos. Embryos were transferred to recipients which were diagnosed as pregnant or open (i.e., non-pregnant) at specific time endpoints (Day-40, Day-62 and birth). Metabolites in CM were analyzed as a function by which to predict pregnancy at each endpoint. We previously showed that metabolites identified and quantified in the CM surrounding embryos, cultured singly, depend on controllable factors, such as culture conditions and embryonic stages at the onset and end of the embryo culture step, as well as, perhaps, the cryopreservation status, since recipients made pregnant with V/W and fresh embryos differ in terms of their metabolic profiles [37,78], probably reflecting the fact that embryos also differ.

Biomarker studies are governed by principles of population science. Thus, the samples analyzed must be sufficient in number and randomness to be representative of the population under study [78,79,80]. Within embryos, random sources refer to different bulls, oocytes from different mothers and, to a certain extent, different embryo production systems, since it is not feasible to adapt each biomarker to the wide assortment of laboratorial conditions in cattle. Thus, although the most valuable biomarkers are those that behave as predictive in populations with high individual variability, discrimination by supervised, controllable, non-random factors can in turn improve the accurateness of predictions. In the case of embryos, discrimination by cryopreservation, breed, culture system and developmental stage can improve the predictions [24,26]. Biomarkers identified in the so-called discovery population increase their value when identified in independent populations and/or sample groups [81,82].

### 4.2. Oocyte Collection and In Vitro Maturation (IVM)

The procedures for in vitro embryo production (IVP) were recently described [1]. In brief, ovaries were collected from slaughtered cows (Matadero de Guarnizo, Spain; Matadero Municipal de Leon, Spain). Antral follicles (3–8 mm diameter) were aspirated and transferred to holding medium (HM) TCM199 (Invitrogen, Barcelona, Spain), 25 mM HEPES and 0.4 mg/mL BSA. Good-quality oocytes (more than three cumulus cell layers and homogenous cytoplasm) were selected for IVM. Cumulus-oocyte complexes (COCs) were rinsed three times in HM and washed three times in maturation medium (MM) consisting of TCM199 NaHCO_3_ (2.2 mg/mL) supplemented with 10% FCS (*v/v*) (F4135), 1.5 μg/mL of porcine FSH-LH (Stimufol; ULg FMV, Liège, Belgium) and 1 μg/mL 17 β-estradiol. COCs (*n* = 30–50) were matured in a four-well dish with 500 μL of MM at 38.7 °C, 5% CO_2_ and high humidity for 22 to 24 h.

### 4.3. In Vitro Fertilization (IVF)

Oocytes were in vitro fertilized (Day-0) with commercial frozen/thawed semen from Asturiana de los Valles (AV) bulls (*n*= 4) and Holstein (*n*= 3) bulls with proven fertility. Motile sperm were obtained following a swim-up protocol [83], incubating for 1 h with pre-equilibrated Sperm-TALP (Tyrode’s albumin lactate pyruvate). Then, the supernatant upper layer, which contained motile sperm, was recovered and centrifuged for 7 min at 200× *g*, and the resultant supernatant was removed. COCs were washed twice in HM and transferred to 4-well dishes containing 500 μL of pre-equilibrated fertilization medium (Fert-TALP) with heparin (10 μg/mL; Calbiochem, La Jolla, CA, USA). COCs and sperm cells (2 × 10^6^ cells/mL) were co-incubated for 18 to 20 h at 38.7 °C in a 5% CO_2_ atmosphere with saturated humidity.

### 4.4. In Vitro Culture (IVC)

Cumulus cells were detached using a vortex, and fertilized oocytes were cultured in modified synthetic oviduct fluid (mSOF) containing 45 μL/mL BME amino acids solution (B6766), 5 μL/mL MEM non-essential amino acids solution (M7145), citrate (0.1 μg/mL), myo-inositol (0.5 μg/mL), and BSA (A3311) (6 mg/mL) with or without 0.1% (*v/v*) FCS (SIGMA F4135), under mineral oil. IVC was carried out in groups (*n* = 35–50) at 38.7 °C, 5% CO_2_, 5% O_2_, 90% N_2_ and saturated humidity until Day-6. On Day-6 (143 h PI) good quality morulae, early blastocysts and blastocysts were selected and cultured individually in 12 µL mSOF with 0.5 mg/mL polyvinyl-alcohol PVA (P8136), without BSA or FCS, under mineral oil for 24 h. On Day-7, embryos at the expanding blastocyst stage (ExB) and fully expanded blastocysts (FEB) were collected and transferred fresh or were frozen. Moreover, Day-7 early blastocysts and blastocysts that had developed from Day-6 morulae were individually re-cultured again for 24 h with new CM and those that reached the FEB stage on Day-8 were frozen, while the remaining embryos were discarded. The SCS in protein-free medium step allows for direct chromatographic analysis of CM for non-invasive studies without previous sample processing, and leads to high pregnancy and birth rates with cryopreserved (vitrified and frozen) and fresh embryos [1,84]. All embryos were transferred to recipients synchronized on cycle Day-7 (168 h PI). Spent CM of each embryo and blank samples (i.e., CM incubated without embryo), collected from the last 24 h of culture, were snap-frozen in LN_2_ and stored at −150 °C until metabolomics analysis.

### 4.5. Embryo Freezing and Thawing

These procedures were recently described in detail [1]. Briefly, ExB and FEB were washed individually three times in PBS + 4 g/L BSA and loaded in freezing medium containing PBS (P4417), 1.5 M EG and 20% CRYO3 (5617, Stem Alpha, St Genis Largentiere, France) for 10 min. Embryos were aspirated in a French straw, loaded between 2 columns with PBS + 0.75 M EG + 20% CRYO3, and 2 further columns PBS + 0.75 M EG + 20% CRYO3 separated by air. The straw was closed with a plug and loaded into a programmable freezer (Crysalis, Cryocontroller PTC-9500; Biogenics INC, Harriman, TN, USA ) at −6 °C for 2 min and seeded once with supercooled forceps. Straws remained for eight further min at −6 °C and were subsequently dehydrated at −0.5 °C/min up to −35 °C. Finally, the straws were stored in LN_2_ until use. For thawing, the straws were held for 10 s in air and 30 s in a bath at 35 °C and carefully dried with 70% ethanol. Each thawed straw with a single embryo was mounted in an ET catheter and directly transferred to recipients.

### 4.6. Recipient Management, Embryo Transfer and Pregnancy Diagnosis

Embryos were transferred to recipient heifers from Asturiana de los Valles (1.74 years), Holstein (1.84 years), and their crosses (1.76 years), in the experimental herd. Recipient traits, feeding, nutrition, management and ET procedures were described in detail [78,85]. Briefly, recipients were synchronized in estrus with an intravaginal progestogen device (PRID Alpha; CEVA, Barcelona, Spain) for 8–11 days, followed by a prostaglandin F_2_α analogue (Dynolitic, Pfizer, Madrid, Spain) injected 48 h before progestogen removal. Blood plasma was collected on Day-0 and Day-7 (before ET) in ethylenediamine tetraacetic acid (EDTA) vacuum tubes via coccygeal vein puncture for progesterone (P4) measurement. An enzyme-linked immunosorbent assay (ELISA) test operating on a 0–40 ng/mL^−1^ scale (EIA-1561, DRG DiagnosticsSpringfield, NJ, USA) was used. The test was sensitive starting from 0.5 ng/mL^−1^, and cross-reactivity from steroids other than P4 was less than 1%. Intra- and inter-assay coefficients of variation were 6% and 7%, respectively.

The criteria for selection of recipients for transfer included observation of standing estrus by experienced carekeepers 2–3 times per day, and heat monitoring with an automated sensor system (Heatphone, Medria, Humeco, Huesca, Spain). In the absence of clear estrous signs, progesterone levels were used to select recipients, with P4 fold change Day-7/Day-0 >8 and Day-7 P4 values >3.5 ng/mL. Before ET, all recipients were clinically examined for detection of a healthy corpus luteum in one ovary by ultrasonography. Pregnancy was diagnosed by ultrasonography on Day-40 and Day-62, and birth rates were monitored.

ETs were performed with fresh and frozen/thawed embryos, non-surgically and under epidural anesthesia. All frozen and transferred embryos were ExB and FEB (*n* = 56), while embryos transferred fresh (*n* = 28) exceptionally included one early blastocyst and one blastocyst. Fresh embryos were washed twice in Embryo Holding Media (019449, IMV Technologies, L’Aigle, France) and mounted in straw in the same medium, while frozen/thawed embryos were directly transferred.

### 4.7. Untargeted Metabolomic Analysis and UHPLC-TOF MS Conditions

All samples were diluted 1:3 (*v/v*) in ultrapure water after being thawed on ice, and directly analyzed in duplicate.

Chromatographic separation (Dionex^TM^ UltiMate 3000 UHPLC, Thermo Fisher Scientific^TM^, Waltham, MA, USA) was achieved using a C18 column (2.1 × 100 mm, 1.8 µm, ACQUITY UPLC^®^ HSS T3, Waters Corp., Milford, MA, USA) in reverse phase (RP) at 30 °C and 250 µL/min total flow rate. Phase A consisted of ultrapure water and phase B acetonitrile, both with 0.1% formic acid (*v/v*). The gradient elution profile, previously validated [28], was as follows: 0 min (0% B), 2 min (0% B), 5 (70% B), 8 min (100% B), 13 min (100% B). The column was equilibrated for 6 min prior to each analysis.

The MS acquisition (Impact-II with conventional ESI ion source, Bruker Daltonics, Billerica, MA, USA) was performed in the positive ionization mode in a scan range from m/z 100 to 1500 and 12 Hz spectra rate. Settings were as follows: nebulizer gas pressure, 2.1 Bar; gas temperature, 300 °C; capillary voltage, 4500 V; drying gas flow rate, 10 L/min MS^2^, obtained from CID fragmentation of the top 3 parent ions of each scan with collision energies ranged from 4 to 20 eV, was used for metabolite identification.

External calibration of the Q-TOF MS using a commercial mixture (ESI Low concentration Tune Mix, Agilent Technologies, Santa Clara, CA, USA) and internal mass calibration with a solution of Na formate clusters (m/z range from 91 to 1383) were carried out as a quality control. Prior to each injection, calibrant was infused at 180 µL/min with a syringe pump during column stabilization to re-calibrate each chromatogram and ensure constant mass accuracy during the whole analysis sequence.

Furthermore, for continuous quality monitoring, a 10 ppb solution of triphenyl phosphate (Sigma Aldrich, St. Luis, MI, USA) was injected every 5 samples to check detector sensitivity, mass accuracy and chromatographic performance. A mix of all sequenced samples was also injected at the beginning and end of the sequence to obtain successful and stable chromatographic resolution of samples. All acquired data were exported by DataAnalysis v.4.2 (Bruker Daltonics, Billerica, MA, USA).

### 4.8. Data Processing

Raw data were extracted and converted to the appropriate format for processing. Data treatment, peak selection, deconvolution, alignment and identification were performed with the software MZmine v2.53 [86] and the R package pRocessomics (https://github.com/Valledor/pRocessomics, accessed on 4 April 2020). Supplementary Table S5 describes the parameters under which data were processed. Data processing included missing value imputation with the Random Forest (RF) algorithm (threshold value = 0.25). Data were filtered using a consistency criterion (0.8 threshold) and abundance balancing using the average of the total intensities of all samples (normalization step). Thereafter, each sample concentration signal was subtracted with their corresponding blank. The resulting output data, with their corresponding retention time, m/z and peak area, were submitted to statistical analysis.

Potential metabolite markers were tentatively identified by matching the obtained MS/MS data to those published in free-access databases: Human Metabolome Database (HMDB), MassBank, GNPS and NIST 14, within a mass accuracy window of 5 ppm.

A total of 118,564 initial variables were obtained after MZmine analysis, and peak areas were then processed using R. Missing value imputation was performed with the Random Forest (RF) algorithm and a threshold value of 0.25. Data were filtered using a consistency criterion (0.8 threshold), and abundance balancing using the average of the total intensities of all samples (normalization step). After processing, a total of 6111 features remained, which were thereafter subtracted with their corresponding blank. The resulting output data were submitted to statistical analysis.

### 4.9. Statistical Analysis

Metabolites in embryo CM were analyzed as a function to predict pregnancy at each pregnancy endpoint (Day-40, Day-62 and birth). The analysis charted the following steps:

#### 4.9.1. Multivariate Statistics

This analysis was performed with Metaboanalyst [87].

We used the discriminant analysis (DA) algorithms sparse Partial Least Square (sPLS-DA) and Orthogonal Partial Least Square (OPLS-DA) for supervised sample separation. Multivariate analysis started by analyzing the entire feature dataset (6111 retained features) as a function of pregnancy at the gestational endpoints D40, D62 and Birth. Subsequently, sample separation was explored by combinations of breed and/or embryonic stages, or their combinations with culture condition and cryopreservation. The effect of each single bull as a random factor was also analyzed.

#### 4.9.2. Block Analyses

A design with fixed, controllable (non-random) factors was used to identify blocks with significant predictive ability, excluding random factors (Metaboanalyst). Thus, blocks consisted of combinations of the fixed factors embryo, culture medium from Day-0 to Day-6 (BSA vs. BSA + FCS) and bull breed (Holstein vs. AV), since each of these factors can alter embryonal metabolism [26]. Furthermore, metabolomics supervised with stage factors improves pregnancy prediction [24]. The block study also included embryo cryopreservation (fresh/F/T) and age of embryo (7/8 days), and analyses were performed at each pregnancy end-point (Day-40, Day-62 and birth). The random factors date (i.e., round of ET performed), bull (*n* = 7) and recipient were not considered within blocks. Two significance levels were used. The first level consisted of Volcano Plots formed by Fold Changes (FCh) >│2│ and *p* < 0.05 or tendencies 0.05 > *p* < 0.10 (parametric and non-parametric statistics by ANOVA and Kruskal–Wallis test, respectively). Metabolites fulfilling such requirements were analyzed at a second level by Receiver Operator Characteristic—area under curve (ROC-AUC) >0.700 and FCh >│2│). The statistical significance (*t*-test) with *p*-value required was <0.10, with minor exceptions to these criteria within blocks having lower sample numbers and the highest ROC-AUCs (0.850–1.000) for specific embryonic transitions for convenience; such low sample sets were only used as complementary information for larger blocks. The study analyzed all factorial combinations from a set with *n* = 84 embryos transferred (*n* = 28 Day-7 fresh; and *n* = 48 Day-7 frozen, and *n* = 8 Day-8 frozen). All embryos (except two fresh) were ExB and FEB transferred to recipients synchronized on cycle Day-7. Only blocks yielding metabolite signals fulfilling the above criteria were described.

#### 4.9.3. Metabolite Identification

Metabolites were first tentatively assigned by comparing very accurate precursor masses to public databases (<10 ppm of difference between measured and exact compound mass), taking advantage of the high resolution of the qTOF instrument. Only those m/zs fulfilling significant conditions in the block study were further validated by using MS/MS data. MS2 spectra were then matched against public databases (Human Metabolome Database (HMDB), MassBank, GNPS and NIST 14, using a 5 ppm threshold) to validate metabolite identification. Different collision energies were applied to each parent mass and resulting spectra were employed for database comparisons. Identifications were only validated when at least the precursor mass and three MS2 ions were coincident.

#### 4.9.4. Univariate Studies in the Whole Dataset

This study investigated how the candidate biomarker metabolites identified in the block study behaved in the entire data set (SAS/STAT; Version 9.2; SAS Institute Inc. Cary, NC, USA). Generalized Linear Models were applied, and Bonferroni correction (*p* < 0.10) was used as a false discovery rate post hoc test. The variable endpoints analyzed were P40, P62, birth, miscarriage after Day-40, and metabolic differences between Day-8 and Day-7 embryos.

#### 4.9.5. Taxonomical Analysis (Class Metabolite Analysis and Block Validation)

Metabolites were grouped in taxonomical classes identified in total blocks at the three developmental endpoints. The overall value of each metabolite as a pregnancy predictor was defined by the numbers of blocks in which the metabolite was significantly predictive (here termed as intrinsic value), and by the number of samples correctly sexed within such blocks (absolute value). Thus, the validation proof of a metabolite as a predictor increases when represented within higher numbers of blocks, different culture and cryopreservation conditions, and increased number of samples correctly identified.

#### 4.9.6. Endpoint Analysis

The endpoint analysis gave an overview on the general impact of metabolites through the pregnancy endpoints, with significant differences in the predictive mode (i.e., early or late pregnancy endpoints) of each metabolite.

#### 4.9.7. Use of Metabolites as Biomarkers

The effectiveness of a metabolite as a pregnancy predictor under specific culture, stage and/or cryopreservation conditions is dependent not only on its ROC-AUC value, but also on the relative abundance of embryonic transitions (i.e., the proportion of each embryonic stage in culture) and culture medium (BSA and BSA + FCS) within blocks represented by each metabolite. Thus, we weighed ROC-AUC with the relative abundance of the group of embryos represented in culture. For this purpose, we used a metadatabase with more embryos (*n* = 1114) than in this study to accurately define the proportions of metabolites expected [26] (see Supplementary Table S6, with the stage distribution abundance). Metabolite biomarkers were used in two ways:

##### Single Biomarker Metabolites

Hits bearing ROC-AUC > 0.700, with which it is possible to accurately predict pregnancy in specific blocks with ≥0.70% effectiveness as single metabolites.

##### Combined Biomarker Metabolites

We combined the predictive power of single metabolites to obtain overall predictions >0.800 within blocks and series. A series is defined by the same conditions for cryopreservation, breed and culture, supported by one or more developmental IC-stages.

## Figures and Tables

**Table 1:** Descriptive pregnancy rates (%) at gestational endpoints Day-40, Day-62 and birth of frozen and fresh in vitro produced embryos arranged by culture conditions from Day-0 to Day-6.

Cryopreservation	Culture	Day	*n*	Day-40	Day-62	Birth
Fresh	BSA	7	17	12 (70.6)	11 (64.7)	9 (52.9)
Fresh	BSA + FCS	7	11	8 (72.7)	8 (72.7)	7/10 (70.0) ^(1)^
Frozen	BSA	7	30	18 (60)	18 (60)	14 (46.7)
		8	8	2 (25)	2 (25)	1 (12.5)
Frozen	BSA + FCS	7	18	11 (61.1)	10 (55.5)	8 (44.4)

^(1)^ One recipient deceased after pregnancy Day-62 and did not reach birth. Day: age of the cultured embryo counted from the onset of in vitro fertilization.

**Table 2:** Probability of changes in relative concentrations of metabolites that differed in culture medium between transferred embryos later diagnosed as pregnant or open at Day-40, Day-62 and birth, and between embryos that miscarried after Day-40 vs. embryos that never reached pregnancy and vs. embryos that reached pregnancy to term.

	Birth		Day-62		Day-40		Miscarriage	
Metabolite	*p*-Value	Bon	*p*-Value	Bon	*p*-Value	Bon	*p*-Value	Bon
l-Leucine	0.0454	0.05						
l-Lysine	0.0494	0.05	0.009	0.05	0.0143	0.05		
Palmitoylethanolamide	0.0782	0.10						
l-Valine			0.0439	0.05	0.0729	0.05		
l-Glutamic acid			0.0740	0.05	0.0844	0.05		
Dimethyl adipate			0.0774	0.05	0.0266	0.05	0.0018	0.05
Lauroyl diethanolamide	0.0112	0.05						
Phosphatidylethanolamine(18:2/20:2)			0.0518	0.05	0.0168	0.05		

GLM analysis parametrized with the following factors: embryo cryopreservation, culture, bull breed, embryonic stages -0 h and 24 h-, and embryonic age. Significant *p*-values are stated as *p* < 0.05, and tendencies 0.05 > *p* < 0.10. Bonferroni (Bon) as a false discovery rate value at levels *p* < 0.05 and *p* < 0.10.

**Table 3:** Taxonomical classification (Classes were lipids—1, amino acids and derivatives—2, benzenoids—3, tri-carboxylic acids—4 and tryptamines and derivatives—5) of metabolites identified as significantly predictive within blocks and pregnancy endpoints D40, D62 and Birth (numbers of blocks with Fz: Frozen embryos; Fh: Fresh embryos; FF: Frozen and Fresh embryos).

		Blocks (N) at Gestational Endpoints											
		Day-40			Day-62			Birth			Total		
Metabolite	Class	Fz	Fh	FF	Fz	Fh	FF	Fz	Fh	FF	Fz	Fh	FF
Citramalic acid	1	1	0	0	0	0	0	1	1	0	2	1	0
5Z-Dodecenoic acid	1	3	0	0	2	0	0	1	0	1	6	0	1
Dimethyl adipate	1	14	0	4	12	1	6	1	1	2	27	2	12
Lauroyl diethanolamide	1	4	0	0	3	0	3	7	1	5	14	1	8
Linoleamide	1	6	0	4	8	0	2	5	0	1	19	0	7
Oleamide	1	2	0	0	3	1	2	0	2	3	5	3	5
Palmitic amide	1	0	0	0	0	0	0	2	1	2	2	1	2
Palmitoylethanolamide	1	1	0	0	0	0	1	7	0	0	8	0	1
Phosphatidylethanolamine(18:2/20:2)	1	4	2	2	3	2	5	4	1	3	11	5	10
MG(16:0/0:0/0:0)	1	1	0	0	1	3	0	12	1	3	14	4	3
12-Hydroxydodecanoic acid	1	9	0	2	11	0	3	14	0	4	34	0	9
Dihydro-alpha-ionone	1	1	0	0	1	0	0	2	0	3	4	0	3
		**60**			**73**			**91**			**224**		
l-Threonine	2	4	0	1	1	0	0	4	0	0	9	0	1
l-Arginine	2	4	0	0	1	0	2	5	0	6	10	0	8
l-Glutamic acid	2	4	1	1	4	1	1	2	4	1	10	6	3
l-Glutamine	2	4	0	1	1	0	3	5	0	3	10	0	7
l-Leucine	2	1	0	0	1	2	0	2	1	1	4	3	1
l-Lysine	2	0	3	1	1	2	3	0	2	1	1	7	5
l-Methionine	2	2	0	1	3	0	0	7	0	3	12	0	4
l-Proline	2	3	0	1	2	0	1	3	0	2	8	0	4
l-Tryptophan	2	0	0	1	1	0	3	0	1	3	1	1	7
l-Tyrosine	2	0	0	1	2	0	2	0	0	2	2	0	5
l-Valine	2	5	1	2	3	0	1	0	0	0	8	1	3
Pipecolic acid	2	0	0	0	2	0	0	1	1	0	3	1	0
l-Histidine	2	2	0	2	2	0	5	3	0	2	7	0	9
l-Phenylalanine	2	1	0	0	1	0	1	1	0	1	3	0	2
Pyroglutamic acid	2	6	0	2	4	0	3	3	0	2	13	0	7
		**55**			**59**			**72**			**186**		
Benzoic acid	3	4	0	1	1	0	1	2	0	4	7	0	6
Indole	3	2	0	2	1	0	3	5	1	2	8	1	7
Phenylacetaldehyde	3	1	0	0	1	1	0	1	3	0	3	4	0
p-Cresol	3	7	0	1	8	0	3	2	0	1	17	0	5
		**18**			**19**			**21**			**58**		
cis-Aconitic acid	4	8	1	1	9	0	1	5	2	2	22	3	4
Citric acid	4	2	2	0	1	2	1	2	1	1	5	5	2
		**14**			**14**			**13**			**41**		
5-Hydroxy-l-tryptophan	5	0	0	0	0	1	0	0	0	1	0	1	1
Cumulative		**147**			**166**			**198**			**511**		

**Table 4:** Single metabolites that predicted pregnancy (Day-40 and Day-62) and birth (Endpoint) with >0.700 coverage within culture medium of IVP embryos transferred to recipients. Each block had specific embryo cryopreservation, bull breed and culture conditions, and is supported by embryonic stages. Embryos were sired by Holstein—H—or Asturiana de los Valles—AV—bulls, and cultured with albumin (BSA) or with albumin + fetal calf serum (FCS) followed by an individual 24 h culture step (IC stage) developmentally defined by embryonic stages at 0 h (M: morula; EB: early blastocyst; B: Blastocyst) and at 24 h (ExB: expanding blastocyst; FEB: Fully expanded blastocyst) that led to embryos aged 7, 8 and both days that were transferred fresh or frozen (Cryo) to Day-7 estrus synchronized recipients. N: samples used to calculate predictions within each metabolite block (P: pregnant; O: Open). Impact: proportions of embryos represented within IC stages developed under each culture conditions. Unfilled cells indicate the independence of this factor. P1: *p*-value by *t*-test; P2: *p*-value by GLM or Kruskal–Wallis test. LogFCh: logarithm of fold change pregnant/non-pregnant metabolite concentration values. AUC: area under curve.

	ROC-Analysis								IC Stage			N		Coverage	
Metabolite	AUC	P1	LogFCh	P2	Endpoint	Cryo	Breed	Culture	0 h	24 h	Age	P	O	Impact	Predicted
Lauroyl diethanolamide	1.000	0.000	−99.000	0.003	Birth	Frozen	AV	BSA				8	5	100.000	100.000
l-Glutamic acid	1.000	0.110	−99.000	0.004	Birth	Fresh	H	BSA				8	4	100.000	100.000
l-Proline	1.000	0.000	−99.000	0.003	P40	Frozen	AV	BSA				9	4	100.000	100.000
l-Proline	1.000	0.000	−99.000	0.003	P62	Frozen	AV	BSA				9	4	100.000	100.000
l-Proline	0.952	0.010	−99.000	0.005	Birth	Frozen	AV	FCS				6	7	100.000	95.238
l-Proline	0.952	0.010	−99.000	0.005	P40	Frozen	AV	FCS				6	7	100.000	95.238
l-Proline	0.952	0.010	−99.000	0.005	P62	Frozen	AV	FCS				6	7	100.000	95.238
l-Methionine	0.944	0.024	−99.000	0.011	P40	Frozen	AV	BSA				9	4	100.000	94.444
l-Methionine	0.944	0.024	−99.000	0.011	P62	Frozen	AV	BSA				9	4	100.000	94.444
Pyroglutamic acid	0.929	0.084	−99.000	0.008	Birth	Frozen	AV	FCS				6	7	100.000	92.857
Pyroglutamic acid	0.929	0.084	−99.000	0.008	P40	Frozen	AV	FCS				6	7	100.000	92.857
Pyroglutamic acid	0.929	0.084	−99.000	0.008	P62	Frozen	AV	FCS				6	7	100.000	92.857
l-Glutamic acid	0.920	0.110	−99.000	0.019	Birth	Fresh	H					10	5	100.000	92.000
l-Arginine	0.905	0.016	−99.000	0.014	Birth	Frozen	AV	FCS				6	7	100.000	90.476
l-Arginine	0.905	0.016	−99.000	0.014	P40	Frozen	AV	FCS				6	7	100.000	90.476
l-Arginine	0.905	0.016	−99.000	0.014	P62	Frozen	AV	FCS				6	7	100.000	90.476
l-Lysine	0.905	0.008	−99.000	0.008	P62	Fresh	AV					7	6	100.000	90.476
l-Threonine	0.905	0.014	−99.000	0.014	Birth	Frozen	AV	FCS				6	7	100.000	90.476
l-Threonine	0.905	0.014	−99.000	0.014	P40	Frozen	AV	FCS				6	7	100.000	90.476
l-Threonine	0.905	0.014	−99.000	0.014	P62	Frozen	AV	FCS				6	7	100.000	90.476
l-Glutamine	0.900	0.021	−99.000	0.003	Birth	Frozen		FCS				8	10	100.000	90.000
l-Methionine	0.900	0.025	−99.000	0.019	Birth	Frozen	AV	BSA				8	5	100.000	90.000
l-Methionine	0.900	0.025	−99.000	0.019	Birth	Frozen	AV	BSA				8	5	100.000	90.000
12-Hydroxydodecanoic acid	0.889	0.029	−99.000	0.035	P40	Frozen	AV	BSA				9	4	100.000	88.889
12-Hydroxydodecanoic acid	0.889	0.029	−99.000	0.035	P62	Frozen	AV	BSA				9	4	100.000	88.889
Benzoic acid	0.889	0.021	−99.000	0.034	P40	Frozen	AV	BSA				9	4	100.000	88.889
Benzoic acid	0.889	0.021	−99.000	0.034	P62	Frozen	AV	BSA				9	4	100.000	88.889
Dihydro-alpha-ionone	0.889	0.019	−99.000	0.036	P40	Frozen	AV	BSA				9	4	100.000	88.889
Dihydro-alpha-ionone	0.889	0.019	−99.000	0.036	P62	Frozen	AV	BSA				9	4	100.000	88.889
l-Lysine	0.889	0.011	−3.512	0.026	Birth	Fresh	AV					6	6	100.000	88.889
Citric acid	0.881	0.069	−99.000	0.022	P62	Fresh	AV					7	6	100.000	88.095
citramalic acid	0.861	0.027	−99.000	0.045	Birth	Fresh	AV					6	6	100.000	86.111
Phenylacetaldehyde	0.860	0.021	−99.000	0.045	Birth	Fresh	H					10	5	100.000	86.000
MG(16:0/0:0/0:0)	0.857	0.062	−99.000	0.035	P62	Fresh	AV					7	6	100.000	85.714
cis-Aconitic acid	0.850	0.023	−99.000	0.047	Birth	Frozen	AV	BSA				8	5	100.000	85.000
Phenylacetaldehyde	0.846	0.070	−99.000	0.082	P62	Fresh	H					13	3	100.000	84.615
l-Histidine	0.833	0.020	−99.000	0.051	Birth	Frozen	AV	FCS				6	7	100.000	83.333
l-Histidine	0.833	0.020	−99.000	0.051	P40	Frozen	AV	FCS				6	7	100.000	83.333
l-Histidine	0.833	0.020	−99.000	0.051	P62	Frozen	AV	FCS				6	7	100.000	83.333
MG(16:0/0:0/0:0)	0.833	0.098	−99.000	0.065	Birth	Fresh	AV					6	6	100.000	83.333
Dimethyl adipate	0.818	0.003	−20.168	0.003	P40	Frozen	AV					15	11	100.000	81.818
Dimethyl adipate	0.818	0.003	−20.168	0.003	P62	Frozen	AV					15	11	100.000	81.818
p-Cresol	0.813	0.050	11.024	0.050	P62	Frozen		FCS			7	10	8	100.000	81.250
Citric acid	0.810	0.085	2.460	0.073	Birth	Frozen	AV	FCS				6	7	100.000	80.952
Citric acid	0.810	0.085	−2.460	0.073	P40	Frozen	AV	FCS				6	7	100.000	80.952
Citric acid	0.810	0.085	−2.460	0.073	P62	Frozen	AV	FCS				6	7	100.000	80.952
Citric acid	0.800	0.043	−99.000	0.093	P40	Fresh	AV					8	4	100.000	80.000
Indole	0.800	0.054	−1.835	0.093	Birth	Frozen	AV	BSA				8	5	100.000	80.000
Indole	0.800	0.054	−1.835	0.093	Birth	Frozen	AV	BSA				8	5	100.000	80.000
l-Lysine	0.800	0.091	3.031	0.093	P40	Fresh	AV					8	5	100.000	80.000
l-Proline	0.800	0.050	−99.000	0.093	Birth	Frozen	AV	BSA				8	5	100.000	80.000
l-Proline	0.800	0.050	−99.000	0.093	Birth	Frozen	AV	BSA				8	5	100.000	80.000
12-Hydroxydodecanoic acid	0.799	0.007	−99.000	0.011	Birth	Frozen	H					9	21	100.000	79.894
5-Hydroxy- l -tryptophan	0.798	0.063	−99.000	0.086	P62	Fresh	AV					7	6	100.000	79.762
l-Valine	0.791	0.014	−22.286	0.014	P40	Frozen	AV					15	11	100.000	79.091
l-Valine	0.791	0.014	−22.286	0.014	P62	Frozen	AV					15	11	100.000	79.091
12-Hydroxydodecanoic acid	0.788	0.003	−99.000	0.003	Birth	Frozen		BSA				15	33	100.000	78.841
Indole	0.786	0.052	−99.000	0.100	Birth	Frozen	AV	FCS				6	7	100.000	78.571
Indole	0.786	0.052	−99.000	0.100	P40	Frozen	AV	FCS				6	7	100.000	78.571
Indole	0.786	0.052	−99.000	0.100	P62	Frozen	AV	FCS				6	7	100.000	78.571
MG(16:0/0:0/0:0)	0.784	0.020	5.508	0.018	P62	Fresh						19	9	100.000	78.363
l-Glutamic acid	0.780	0.002	−99.000	0.004	P40	Fresh	AV			ExB + FEB		8	3	100.000	77.950
p-Cresol	0.771	0.045	11.457	0.045	P40	Frozen	AV				7	15	8	100.000	77.083
p-Cresol	0.771	0.045	11.457	0.045	P62	Frozen	AV				7	15	8	100.000	77.083
l -Leucine	0.770	0.031	−99.000	0.017	P62	Fresh						19	9	100.000	77.005
l-Glutamic acid	0.762	0.018	−14.573	0.018	P62	Fresh	AV				7	7	6	100.000	76.190
l-Glutamine	0.762	0.033	15.617	0.033	Birth	Frozen	AV				7	14	9	100.000	76.190
12-Hydroxydodecanoic acid	0.762	0.017	−99.000	0.049	Birth	Frozen	H	BSA				7	18	100.000	76.190
MG(16:0/0:0/0:0)	0.759	0.026	5.619	0.026	P62	Fresh				ExB + FEB		19	7	100.000	75.940
Citric acid	0.759	0.033	−99.000	0.024	P62	Fresh						19	9	100.000	75.936
Dimethyl adipate	0.756	0.017	17.092	0.017	Birth	Frozen	AV					14	12	100.000	75.595
Dimethyl adipate	0.750	0.054	−18.047	0.054	P40	Frozen	AV				7	15	8	100.000	75.000
Lauroyl diethanolamide	0.750	0.006	−9.767	0.006	Birth	Frozen	AV					14	12	100.000	75.000
l-Leucine	0.750	0.065	−99.000	0.065	P62	Fresh	AV					7	6	100.000	75.000
Indole	0.744	0.017	−99.000	0.037	Birth	Frozen	AV					14	12	100.000	74.405
cis-Aconitic acid	0.739	0.026	−21.627	0.026	P40	Frozen	AV					15	11	100.000	73.939
Pyroglutamic acid	0.739	0.060	−99.000	0.041	P40	Frozen	AV					15	11	100.000	73.939
Pyroglutamic acid	0.739	0.060	−99.000	0.041	P62	Frozen	AV					15	11	100.000	73.939
l-Methionine	0.739	0.034	−99.000	0.013	Birth	Frozen		BSA				15	33	100.000	73.913
cis-Aconitic acid	0.738	0.014	−16.641	0.014	P40	Frozen		BSA			7	18	12	100.000	73.843
l-Glutamic acid	0.738	0.043	8.994	0.043	Birth	Frozen	AV					14	12	100.000	73.810
Phosphatidylethanolamine(18:2/20:2)	0.738	0.094	−99.000	0.090	Birth	Frozen		FCS				8	10	100.000	73.750
l-Valine	0.736	0.006	4.109	0.018	P40	Fresh	AV			ExB + FEB		8	3	100.000	73.602
cis-Aconitic acid	0.733	0.015	−99.000	0.020	P40	Fresh	AV			ExB + FEB		8	3	100.000	73.292
Palmitoylethanolamide	0.730	0.086	−99.000	0.085	Birth	Frozen	H	BSA				7	18	100.000	73.016
l-Glutamine	0.725	0.064	−13.100	0.064	P40	Frozen	AV				7	15	8	100.000	72.500
l -Methionine	0.720	0.064	−99.000	0.063	Birth	Frozen	H					9	21	100.000	71.958
Oleamide	1.000	0.011	3.786	0.009	P62	Fresh		BSA		FEB		6	3	71.490	71.490
Lauroyl diethanolamide	0.713	0.022	−99.000	0.022	Birth	Frozen		BSA				15	33	100.000	71.304
Citric acid	0.713	0.052	2.972	0.088	P40	Fresh						20	8	100.000	71.250
Indole	0.706	0.011	−4.439	0.032	Birth		AV					20	18	100.000	70.556
l-Lysine	0.705	0.011	3.605	0.040	P40	Fresh	AV			ExB + FEB		8	3	100.000	70.497
l-Threonine	0.702	0.037	−99.000	0.085	Birth	Frozen	AV					14	12	100.000	70.238
Phosphatidylethanolamine(18:2/20:2)	0.700	0.055	−5.441	0.055	P40	Frozen	AV				7	15	8	100.000	70.000

**Table 5:** Best metabolite block combinations (combined coverage) that predicted pregnancy (Day-40 and Day-62 and birth) (Endpoint) with >0.800 coverage within IVP embryos transferred to recipients. Each series consists of blocks with the same embryo cryopreservation, bull breed and culture conditions, and supported by embryonic stages. IVP embryos (sired by Holstein—H—or Asturiana de los Valles—AV—bulls) were cultured with albumin (BSA) or with albumin + fetal calf serum (FCS) followed by a single 24 h culture step (IC stage) developmentally defined by embryonic stage at 0 h (M: morula; EB: early blastocyst; B: Blastocyst) and at 24 h (ExB: expanding blastocyst; FEB: fully expanded blastocyst) that led to embryos aged 7 or 8 days that were transferred fresh or frozen (Cryo) to Day-7 estrus synchronized recipients. N: samples used to calculate predictions within each metabolite block (P: pregnant; O: Open). Impact: proportion of embryos represented within IC stages developed under each culture conditions. No filled data indicates the independence of this factor. Asterisks indicate blocks aggregated within a series to form the combined average value that is shown below the respective asterisks.

							IC Stage			N		Single Coverage		Combined
Series	Metabolite	Class	Endpoint	Cryo	Breed	Culture	0 h	24 h	Age	P	O	Impact	Predicted	Coverage
1	p-Cresol	3	P40	Frozen	H		EB + B			11	10	56.371	44.071	*
1	citramalic acid	1	P40	Frozen	H		M			5	4	43.630	39.267	83.3385
														
2	Dimethyl adipate	1	P40	Frozen			EB + B			21	20	56.371	47.781	*
2	Linoleamide	1	P40	Frozen			M			10	5	43.630	38.394	86.1751
														
3	l -Proline	2	P40				EB + B	ExB		8	2	15.871	12.998	*
3	Linoleamide	1	P40				EB + B	FEB		28	23	40.500	34.977	*
3	Linoleamide	1	P40				M	ExB		8	3	20.080	18.825	*
3	l -Tyrosine	2	P40				M	FEB		7	3	23.550	23.550	90.3504
														
4	12-Hydroxydodecanoic acid	1	P62	Frozen		BSA	EB	FEB		3	8	23.770	22.780	*
4	Pyroglutamic acid	2	P62	Frozen		BSA	B	FEB		10	6	10.790	9.531	*
4	l -Tyrosine	2	P62	Frozen		BSA	M	FEB		6	3	36.930	36.930	*
4	l -Histidine	2	P62	Frozen		BSA		ExB		7	3	28.510	25.795	95.0353
														
5	Dimethyl adipate	1	P62	Frozen			EB			6	13	41.470	35.090	*
5	Phosphatidylethanolamine(18:2/20:2)	1	P62	Frozen			B		7	13	6	14.900	11.462	*
5	Linoleamide	1	P62	Frozen			M			4	3	43.630	38.176	84.7278
6	Palmitoylethanolamide	1	P62			BSA		ExB		7	3	28.510	28.510	*
6	Dimethyl adipate	1	P62			BSA		FEB		24	19	71.490	53.539	82.0489
7	Pyroglutamic acid	2	P62			FCS		ExB		8	3	43.380	37.958	*
7	l -Glutamine/D-Glutamine	2	P62			FCS		FEB		10	8	56.620	49.543	87.5000
8	Dimethyl adipate	1	P62				EB			15	16	41.470	29.374	*
8	12-Hydroxydodecanoic acid	1	P62				B			20	10	14.900	10.430	*
8	l -Lysine	2	P62				M	ExB		7	4	20.080	17.211	*
8	l -Tyrosine	2	P62				M	FEB		7	3	23.550	23.550	80.5658
9	citramalic acid	1	Birth	Frozen	H		M			5	4	43.630	39.267	87.3472
9	MG(16:0/0:0/0:0)	1	Birth	Frozen	H		EB + B			4	17	56.370	48.080	*
														
10	12-Hydroxydodecanoic acid	1	Birth	Frozen		BSA	EB + B			9	19	42.992	36.956	*
10	Pipecolic acid	2	Birth	Frozen		BSA	M			6	4	57.010	46.321	83.2765
														
11	Pyroglutamic acid	2	Birth	Frozen			M			10	5	43.628	34.902	*
11	12-Hydroxydodecanoic acid	1	Birth	Frozen			EB + B			13	28	56.370	46.924	81.8259
12	l -Glutamic acid	2	Birth	Fresh	H		EB + B			8	5	56.370	50.733	*
12	L-Lysine	2	Birth	Fresh			M			4	4	43.630	40.903	91.6361
13	l -Glutamic acid	2	Birth	Fresh		BSA	EB + B			6	4	42.990	42.990	*
13	Indole	3	Birth	Fresh		BSA	M			3	4	57.010	57.010	100.0000
14	cis-Aconitic acid	4	Birth	Fresh		BSA		FEB		5	4	71.490	64.341	*
14	Phenylacetaldehyde	3	Birth	Fresh		BSA		ExB + B6		4	3	28.510	28.510	92.8510
15	Oleamide	1	Birth			BSA	EB			8	11	29.684	25.299	*
15	cis-Aconitic acid	4	Birth			BSA	B			7	12	13.308	12.199	*
15	Indole	3	Birth			BSA	M	ExB		3	5	20.079	20.079	*
15	l -Tyrosine	2	Birth			BSA	M	FEB		6	3	36.927	36.927	94.5039
16	Citric acid	4	Birth			FCS		ExB		7	4	43.381	37.184	*
16	l -Glutamine	2	Birth			FCS		FEB		8	9	56.618	49.541	86.7245
17	l -Proline	2	Birth				EB	ExB		4	4	12.610	11.034	*
17	Dimethyl adipate	1	Birth				EB	FEB		8	15	28.863	23.090	*
17	l -Methionine	2	Birth				B			13	16	14.898	11.531	*
17	l -Glutamine	2	Birth				M	ExB		7	4	20.075	18.068	*
17	l -Tyrosine	2	Birth				M	FEB		6	4	23.553	23.553	87.2759

Taxonomical classes (Class): (1) Lipids; (2) Amino acids; (3) Benzenoids; (4) Carboxylic acids; (5) Tryptamines and derivatives.

## Data Availability

The data presented in this study are available on reasonable request from the corresponding author. The data are not publicly available due to privacy and/or ethical concerns.

## Supplementary Materials

The following are available online at https://www.mdpi.com/article/10.3390/metabo11080484/s1, Table S1: Summary of embryos (sample n°) under study, including culture from Day-0 to Day-6 (BSA or BSA + FCS), cryopreservation status (Cryo), embryonic stages before (Day-6) and after (Day-7) individual culture period, age of the embryo (days), bull breed (AV: Asturiana de los Valles) and progress through the diagnosed gestational endpoints, Table S2: Peak identification, m/z, retention time and database of the annotated metabolites in the study after comparison between MS/MS fragments to available metabolite databases using SIRIUS v4.4.29. Details of fragmentation trees, molecular formula identification, median mass error (ppm) of explained peaks and CSI: FingerID are shown. Metabolites with different RT but the same annotation, and the two adducts identified for L-Arginine, were considered together in the biomarker analysis. Three of the fragments detected for each metabolite are also shown. Validation: metabolites with annotation confirmed in the MS3 analysis. Explained peaks: number of peaks in the spectrum which can be explained by the fragmentation tree. Similarity: numbers in percentage between the predicted fingerprint and the fingerprint of each candidate, Table S3: General results of metabolomic block analysis consisting of Receiver Operator Characteristic (ROC)-analysis (defined by Area Under Curve –AUC– > 0.700, *p*-value by *t*-test < 0.10 and fold change │2│ (written as LogFCh) concentrations between embryos that led to non-pregnant/pregnant recipients) combined with GLM or Kruskal–Wallis (KW) test *p*-value < 0.10. Minor exceptions to these criteria were used within blocks with lower sample numbers and only the highest ROC-AUCs (0.850–1.000) for specific embryonic transitions; such blocks were only used as complementary information (*p*-values shown in bold). Pregnancies were diagnosed at specific gestational times (Endpoint) within embryos obtained from oocytes fertilized in vitro with Asturiana de los Valles (AV) or Holstein (H) bulls, and cultured from 6 days or 7 days in synthetic oviduct fluid medium supplemented with albumin (BSA) or with albumin + fetal calf serum (FCS) followed by a single 24 h culture step developmentally defined by embryonic stage at 0 h (M: morula; EB: early blastocyst; B: Blastocyst) and at 24 h (ExB: expanding blastocyst; FEB: Fully expanded blastocyst) that led to embryos aged (Age) 7 or 8 days that were transferred fresh or frozen (Cryo) to Day-7 estrus synchronized recipients. Each line represents a block in which a metabolite concentration significantly discriminates between pregnant and non-pregnant recipients under each condition, numerically represented by the impact of embryos under the defined culture, stage and age conditions as a proportion of the total embryos, leading to a proportion of embryos in culture predicted for pregnancy by such a metabolite. The effect of embryonic sex within such culture condition or block (not explored in this work) was defined from reviewing Gimeno et al., 2021 (submitted) in a specific, larger study for identification purposes. Taxonomical classes (Class): (1) Lipids; (2) Amino acids; (3) Benzenoids; (4) Carboxylic acids; (5) Tryptamines and derivatives, Table S4: Metabolites quantified in embryo culture medium that, in combinations, predicted pregnancy (Day-40 and Day-62) and birth (Endpoint) with ROC-AUC > 0.800 within embryos obtained from oocytes fertilized in vitro with Asturiana de los Valles (AV) or Holstein (H) bulls, and cultured 6 days or 7 days in synthetic oviduct fluid medium supplemented with albumin (BSA) or with albumin + fetal calf serum (FCS) followed by a single 24 h culture step (IC stage) developmentally defined by embryonic stage at 0 h (M: morula; EB: early blastocyst; B: Blastocyst) and at 24 h (ExB: expanding blastocyst; FEB: Fully expanded blastocyst) that led to embryos aged (Age) 7 or 8 days that were transferred fresh or frozen (Cryo) to Day-7 estrus-synchronized recipients. Block combinations are shown arranged in series with the same embryo cryopreservation, bull breed and culture conditions, and supported by embryonic stages. Each block fulfilled the criteria of FCh >│2│ and significant *p* < 0.05 or tendencies 0.05 > *p* < 0.10 (parametric and non-parametric statistics by ANOVA and Kruskal–Wallis test) and ROC-AUC > 0.800 with significant *t*-test *p* < 0.05 or tendencies 0.05 > *p* < 0.10. For convenience, minor exceptions to these criteria within blocks with lower sample numbers and only the highest ROC-AUCs (0.850–1.000) for specific embryonic transitions (*p*-values highlighted in yellow). Each metabolite within a block shows “single coverage”, defined by the percentage frequency of such embryonic transition (i.e., “Impact”, obtained within a larger dataset from Gimeno et al., (2021) and the “Predicted value” (i.e., Impact*ROC-AUC). The combined coverage adds up single impacts of metabolites (upper squares marked with asterisks) to give a total coverage, (values ranked from the highest predictive combination –Predicted 1- and following ones –Predicted 2, Predicted 3–, on the right). Colored lines reflect one of the highest predictive metabolite combinations within a series, although any other metabolite in the table can also be combined to give predictive values >0.800. Taxonomical classes (Class): (1) Lipids; (2) Amino acids; (3) Benzenoids; (4) Carboxylic acids; (5) Tryptamines and derivatives, Table S5: Chromatography and Mzmine data processing workflow with details of steps, parameters and values used, Table S6: Descriptive percent distribution of transitions between Day-6 embryonic stages (M: morula; EB: early blastocyst; B: blastocyst) to Day-7 expanding blastocyst (ExB) and fully expanded blastocysts (FEB) after Day-0 to Day-6 culture with BSA or BSA + FCS).
