# Supplementary material for: The Metabolic Signature of In Vitro Produced Bovine Embryos Helps Predict Pregnancy and Birth after Embryo Transfer
Source: Metabolites. 2021 Jul 27;11(8):484. doi: 10.3390/metabo11080484 (PMC8399324; doi:10.3390/metabo11080484)
Supplement: Supplementary file 1 [file metabolites-11-00484-s001.zip › metabolites-1315068-supplementary/SUPPLEMENTARY TABLE 1 - SAMPLES in study 280621.pdf]

Supplementary Table 1

Summary of embryos (sample nº) under study, including culture from Day-0 to Day-6 (BSA or BSA+FCS), cryopreservation status (Cryo), embryonic stages before (Day-6) and after (Day-7) individual culture period, age of the embryo (days), bull breed (AV: Asturiana de los Valles) and progress through the diagnosed gestational endpoints.

			Stage				Gestational status		
Sample	Culture	Cryo	Day-6	Day-7	Age	Bull breed	Day-40	Day-62	Term
1473	BSA	Fresh	M	B	7	AV	Open	Open	No Birth
1487	BSA	Fresh	M	ExB	7	AV	Pregnant	Pregnant	Birth
1488	BSA	Fresh	M	EB	7	AV	Open	Open	No Birth
1509	BSA+FCS	Fresh	M	ExB	7	AV	Pregnant	Pregnant	Birth
1519	BSA	Frozen	B	FEB	7	AV	Pregnant	Pregnant	Birth
1520	BSA	Frozen	B	FEB	7	AV	Pregnant	Pregnant	Birth
1521	BSA	Frozen	B	FEB	7	AV	Pregnant	Pregnant	Birth
1522	BSA	Frozen	M	FEB	7	AV	Pregnant	Pregnant	Birth
1523	BSA	Frozen	M	FEB	7	AV	Pregnant	Pregnant	Birth
1571	BSA+FCS	Fresh	EB	FEB	7	AV	Open	Open	No Birth
1572	BSA+FCS	Fresh	B	ExB	7	AV	Pregnant	Pregnant	Birth
1613	BSA	Frozen	EB	FEB	7	Holstein	Open	Open	No Birth
1614	BSA	Frozen	M	ExB	7	Holstein	Pregnant	Pregnant	Birth
1615	BSA	Frozen	M	FEB	7	Holstein	Pregnant	Pregnant	Birth
1675	BSA	Frozen	B	FEB	7	Holstein	Pregnant	Pregnant	No Birth
1678	BSA	Frozen	M	ExB	7	Holstein	Open	Open	No Birth
1679	BSA	Frozen	M	FEB	7	Holstein	Open	Open	No Birth
1680	BSA	Frozen	M	FEB	7	Holstein	Pregnant	Pregnant	Birth
1681	BSA	Frozen	M	FEB	7	Holstein	Open	Open	No Birth

1725	BSA	Frozen	B	FEB	7	Holstein	Open	Open	No Birth
1732	BSA+FCS	Frozen	B	FEB	7	Holstein	Pregnant	Open	No Birth
1757	BSA+FCS	Fresh	B	FEB	7	Holstein	Pregnant	Pregnant	Birth
1758	BSA+FCS	Fresh	B	FEB	7	Holstein	Pregnant	Pregnant	Birth
1762	BSA	Frozen	B	FEB	7	Holstein	Pregnant	Pregnant	No Birth
1773	BSA	Fresh	EB	FEB	7	Holstein	Open	Open	No Birth
1782	BSA+FCS	Frozen	B	FEB	7	AV	Pregnant	Pregnant	Birth
1783	BSA+FCS	Frozen	EB	FEB	7	AV	Open	Open	No Birth
1784	BSA+FCS	Frozen	EB	FEB	7	AV	Open	Open	No Birth
1785	BSA+FCS	Frozen	M	FEB	7	AV	Pregnant	Pregnant	Birth
1786	BSA+FCS	Frozen	M	ExB	7	AV	Pregnant	Pregnant	Birth
1790	BSA+FCS	Frozen	B	FEB	7	Holstein	Pregnant	Pregnant	No Birth
1799	BSA	Frozen	B	FEB	7	Holstein	Open	Open	No Birth
1842	BSA+FCS	Frozen	M	ExB	7	AV	Open	Open	No Birth
1846	BSA+FCS	Frozen	B	FEB	7	AV	Pregnant	Pregnant	Birth
1847	BSA+FCS	Frozen	B	FEB	7	AV	Pregnant	Pregnant	Birth
1848	BSA+FCS	Frozen	EB	FEB	7	AV	Pregnant	Pregnant	Birth
1900	BSA+FCS	Frozen	M	ExB	7	Holstein	Pregnant	Pregnant	Birth
1901	BSA+FCS	Frozen	M	ExB	7	Holstein	Pregnant	Pregnant	Birth
1905	BSA	Frozen	M	ExB	7	Holstein	Open	Open	No Birth
1993	BSA+FCS	Fresh	B	FEB	7	AV	Open	Open	No Birth
1994	BSA+FCS	Fresh	EB	ExB	7	AV	Pregnant	Pregnant	Birth
2041	BSA+FCS	Frozen	EB	ExB	7	Holstein	Pregnant	Pregnant	No Birth
2142	BSA+FCS	Fresh	EB	ExB	7	Holstein	Open	Open	No Birth
2143	BSA	Fresh	M	ExB	7	Holstein	Pregnant	Pregnant	Birth
2175	BSA	Frozen	M	FEB	7	AV	Pregnant	Pregnant	Birth

2176	BSA+FCS	Frozen	EB	ExB	7	AV	Open	Open	No Birth
2177	BSA+FCS	Frozen	EB	FEB	7	AV	Open	Open	No Birth
2178	BSA+FCS	Frozen	EB	FEB	7	AV	Open	Open	No Birth
2179	BSA+FCS	Frozen	B	FEB	7	AV	Open	Open	No Birth
2242	BSA	Frozen	B	FEB	7	Holstein	Pregnant	Pregnant	Birth
2283	BSA	Frozen	EB	FEB	7	Holstein	Open	Open	No Birth
2284	BSA	Frozen	EB	FEB	7	Holstein	Open	Open	No Birth
2285	BSA	Frozen	B	FEB	7	Holstein	Open	Open	No Birth
2294	BSA	Frozen	EB	FEB	8	Holstein	Pregnant	Pregnant	Birth
2295	BSA	Frozen	EB	FEB	8	Holstein	Open	Open	No Birth
2296	BSA	Frozen	EB	FEB	7	Holstein	Pregnant	Pregnant	Birth
2297	BSA	Frozen	B	FEB	8	Holstein	Open	Open	No Birth
2298	BSA	Frozen	B	FEB	8	Holstein	Pregnant	Pregnant	No Birth
2308	BSA	Fresh	M	ExB	7	AV	Pregnant	Open	No Birth
2309	BSA	Fresh	M	FEB	7	AV	Open	Open	No Birth
2319	BSA	Frozen	EB	ExB	7	Holstein	Pregnant	Pregnant	Birth
2384	BSA	Fresh	EB	FEB	7	Holstein	Pregnant	Pregnant	Birth
2385	BSA	Fresh	EB	ExB	7	Holstein	Pregnant	Pregnant	No Birth
2386	BSA	Fresh	B	FEB	7	Holstein	Open	Open	No Birth
2437	BSA	Fresh	EB	FEB	7	Holstein	Pregnant	Pregnant	Birth
2438	BSA	Fresh	EB	FEB	7	Holstein	Pregnant	Pregnant	Birth
2439	BSA	Fresh	M	FEB	7	Holstein	Pregnant	Pregnant	Birth
2454	BSA	Fresh	EB	FEB	7	Holstein	Pregnant	Pregnant	Birth
2456	BSA	Fresh	B	FEB	7	Holstein	Pregnant	Pregnant	No Birth
2474	BSA	Frozen	B	FEB	7	AV	Open	Open	No Birth
2489	BSA	Frozen	B	FEB	8	AV	Open	Open	No Birth

2499	BSA	Frozen	EB	FEB	8	AV	Open	Open	No Birth
2500	BSA	Frozen	EB	FEB	8	AV	Open	Open	No Birth
2504	BSA+FCS	Fresh	EB	ExB	7	AV	Pregnant	Pregnant	Birth
2505	BSA+FCS	Fresh	EB	FEB	7	AV	Pregnant	Pregnant	Birth
2506	BSA+FCS	Fresh	B	FEB	7	AV	Pregnant	Pregnant	**Deceased**
2516	BSA	Frozen	EB	FEB	7	AV	Pregnant	Pregnant	No Birth
2517	BSA	Frozen	B	FEB	7	AV	Pregnant	Pregnant	Birth
2518	BSA	Frozen	B	FEB	7	AV	Pregnant	Pregnant	Birth
2551	BSA	Frozen	B	FEB	7	Holstein	Pregnant	Pregnant	No Birth
2557	BSA	Frozen	EB	FEB	7	Holstein	Open	Open	No Birth
2563	BSA	Frozen	EB	FEB	8	Holstein	Open	Open	No Birth
2575	BSA	Fresh	B	ExB	7	Holstein	Pregnant	Pregnant	Birth
2576	BSA	Fresh	EB	ExB	7	Holstein	Pregnant	Pregnant	Birth

M: Morula; EB: early blastocyst; B: blastocyst; ExB: expanded blastocyst; FEB: fully expanded blastocyst.

The recipient pregnant of the embryo NO 2506 had a sudden death after the pregnancy Day-62.
