# Supplementary material for: The Metabolic Signature of In Vitro Produced Bovine Embryos Helps Predict Pregnancy and Birth after Embryo Transfer
Source: Metabolites. 2021 Jul 27;11(8):484. doi: 10.3390/metabo11080484 (PMC8399324; doi:10.3390/metabo11080484)
Supplement: Supplementary file 1 [file metabolites-11-00484-s001.zip › metabolites-1315068-supplementary/SUPPLEMENTARY TABLE 5- uhplc parameters 040721.pdf]

Supplementary Table 5

Chromatography and Mzmine data processing workflow with details of steps, parameters and values used

**Steps**	**Parameters**	**Values**
1. Mass detection<br>Mass detector: Centroid	RT Range	0-12 MIN
	Noise level	1.3E2
		1.5E2
2. ADAP chromatogram builder	Min group size in # of scans	5
	Group intensity threshold	5.0E2
	Min highest intensity	1.3E2
		1.5E2
	m/z tolerance	0.005m/z – 15ppm
	RT	0-12min
3. Smoothing	Filter width	5
		7
4. Deconvolution<br>Wavelets (ADAP)<br>m/z center calculation: MEDIAN	S/N threshold	7
	S/N estimator	Intensity window SN
	Min feature height	1.3E2
	Coefficient/área threshold	100
	Peak duration range	0-10
	RT wavelet range	0-0.1
5. Filtering	Peak filter<br>-Duration	0.00-0.85
6. Alignment	Join Aligner	

	m/z tolerance	0.005 m/z – 5.0 ppm
	Weight for m/z	90
	RT tolerance	0.5 min
	Weight for RT	10
