# Supplementary material for: The Metabolic Signature of In Vitro Produced Bovine Embryos Helps Predict Pregnancy and Birth after Embryo Transfer
Source: Metabolites. 2021 Jul 27;11(8):484. doi: 10.3390/metabo11080484 (PMC8399324; doi:10.3390/metabo11080484)
Supplement: Supplementary file 1 [file metabolites-11-00484-s001.zip › metabolites-1315068-supplementary/SUPPLEMENTARY TABLE 6 - EMBRYONIC STAGE DISTRIBUTION 040721.pdf]

Supplementary Table 6

Descriptive percent distribution of transitions between Day-6 embryonic stages (M: morula; EB: early blastocyst; B: blastocyst) to Day 7expanding blastocyst (ExB) and fully expanded blastocysts (FEB) after Day-0 to Day-6 culture with BSA or BSA+FCS).

	BSA (% Day-7)			FCS (% Day-7)		
Day-6	N	ExB	FEB	N	ExB	FEB
B	27	2.514	10.794	43	4.008	12.480
EB	71	5.917	23.768	161	19.303	33.959
Morula	464	20.080	36.928	348	20.071	10.179
Total	562	100 %		552	100 %	

Data taken from Gimeno et al 2021 (submitted)
